# *In vitro* assessment of antitumor activities of the PI3K/mTOR inhibitor GSK2126458

**DOI:** 10.1186/s12935-014-0090-z

**Published:** 2014-09-24

**Authors:** Alia Albawardi, Muna Al Ayyan, Mohamed Al Bashir, Abdul-Kader Souid, Saeeda Almarzooqi

**Affiliations:** Department of Pathology, College of Medicine & Health Sciences, United Arab Emirates University, P.O. Box: 17666, Al-Ain, United Arab Emirates; Surgery Department, Tawam Hospital, Al Ain, United Arab Emirates; Department of Surgery, Tawam Hospital, Al Ain, United Arab Emirates; Department of Surgery, College of Medicine & Health Sciences, United Arab Emirates University, Al Ain, United Arab Emirates; Department of Pediatrics, College of Medicine and Health Sciences, United Arab Emirates University, P.O. Box 17666, Al Ain, United Arab Emirates

**Keywords:** GSK2126458, PI3K, mTOR, Cytotoxicity, Caspases, Cellular respiration

## Abstract

**Background:**

Up-regulation of the PI3K/mTOR (phosphatidylinositol-3′ kinase/mammalian target of rapamycin) signaling is common in carcinoma. Consistently, targeting these molecules has been shown to halt the growth of many tumors. The main purpose of this study was to develop surrogate biomarkers of the antitumor activity of PI3K/mTOR inhibitors.

**Methods:**

Fragments from eight tumors were collected immediately after resection in ice-cold RPMI gassed with 95% O_2_ :5% CO_2_. Viability was determined by measuring tumor cellular respiration (mitochondrial O_2_ consumption). The specimens were incubated at 37°C with and without 50 nM GSK2126458 (a highly potent and selective inhibitor of PI3K/mTOR) for 90 min. The tissue was then processed for histology, measurement of intracellular caspase-3 activity (using the caspase-3 substrate *N*-acetyl-asp-glu-val-asp-7-amino-4-methylcoumarin), and immunohistochemical detection of the apoptotic biomarkers caspase-3, cytochrome C, and annexin A2.

**Results:**

GSK2126458 induced morphologic changes in four tumors (two invasive ductal carcinomas, one invasive lobular carcinoma, and one ovarian dysgerminoma), intracellular caspase-3 activity in three tumors (two invasive ductal carcinomas and one poorly differentiated signet ring adenocarcinoma of gastric origin), and immunohistochemical evidence of apoptosis in at least four tumors (three invasive ductal carcinomas and one adenocarcinoma of gastric origin). Two tumors (ovarian serous carcinoma and moderately differentiated adenocarcinoma of colorectal origin) demonstrated no treatment effect.

**Conclusion:**

These preliminary results demonstrate the feasibility of using *in vitro* biomarkers for detecting antitumor activities of the rapidly emerging PI3K/mTOR inhibitors.

**Electronic supplementary material:**

The online version of this article (doi:10.1186/s12935-014-0090-z) contains supplementary material, which is available to authorized users.

## Background

GSK2126458, a highly potent and selective inhibitor of class I phosphoinositide 3-kinase (PI3K) and mammalian target of rapamycin 1/2 complexes (mTOR) [1,2], is in clinical trials for treatment of various solid tumors [3]. This agent targets the critical survival pathway PI3K/PTEN/Akt/mTOR [4,5]. Inhibiting these signals has been shown to promote apoptosis in tumor cells and impair cellular bioenergetics (the biochemical processes involved in energy biotransformation, such as oxidative phosphorylation or cellular respiration) [6,7].

PI3K is activated by growth factor-stimulated receptor tyrosine kinases, leading to downstream activation of Akt and mTOR with phosphorylation of key substrates involved in preventing cell death [8]. These signals are highly triggered in many cancer types; and mutations in PI3K/AKT have been implicated in the pathogenesis of several human carcinomas [9]. PI3K activating mutations, for example, are frequently reported in breast cancer [10]. Consistently, inhibitors of PI3K possess potent activities in these tumors [11]; and many of these drugs are in clinical trials with promising results [12,13].

Recently an *in vitro* method suitable for performing toxicological studies on thin slices of murine tissue was described [14-17]. Adverse effects of agents were assessed by histology and measurements of intracellular caspase activity. A similar approach is employed here to assess *in vitro* activities of GSK2126458 in tumors studied immediately after resection. Morphologic and apoptotic changes are documented in tumors treated with 50 nM GSK2126458 for 90 min [12]. These preliminary results may stimulate further development of rapid *in vitro* assays for assessing sensitivity of tumors to PI3K/PTEN/Akt/mTOR inhibitors.

## Results

**Figure 1:**
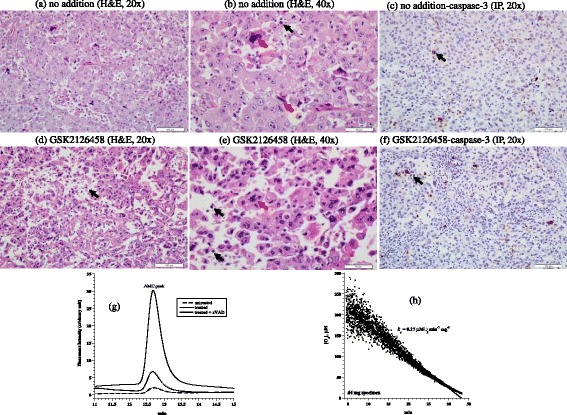
**Invasive ductal carcinoma. (a-f)** Histology and expression of caspase-3 by immunoperoxidase. **(a-b)** Untreated tumor demonstrating sheets of cohesive neoplastic cells demonstrating apoptotic bodies (black arrow) and mitotic figures (red arrow), H&E at 20× and 40×, respectively. **(d-e)** Treated tumor demonstrating loss of cellular cohesion and increased apoptotic bodies (black arrow), H&E at 20× and 40×, respectively. **(c)** Untreated tumor demonstrating positivity (black arrow) for caspase-3 in up to 10% of neoplastic cells compared to 20% in treated tumor **(f)**, immunoperoxidase (IP), 20×. **(g)** HPLC runs of intracellular caspase-3 activity in treated and untreated tumor specimens. The AMC peak (retention time, ~12.8 min) in treated tumor was blocked by the pancaspase inhibitor zVAD, confirming cleavage of Ac-DEVD-AMC was mediated by caspases. **(h)** Cellular respiration, measured on tumor arrival to the laboratory to affirm viability.

Clinicopathological features of the eight studied tumors are listed in Table 1. The first tumor was invasive ductal carcinoma of the breast (Nottingham histological grade 3). It was positive for estrogen receptor (ER+), negative for progesterone receptor (PR-), expressed human epidermal growth factor receptor 2-neu (Her2-neu+), and had a Ki-67 proliferation index of 70%. Histological features of untreated tumor revealed pleomorphic neoplastic cells arranged in cohesive nests and sheets with numerous mitotic figures (Figure 1a-b). Treated tumor revealed decreased cellular density and increased disintegration of neoplastic cells with numerous apoptotic bodies (Figure 1d-e). Expression of caspase-3 by immunoperoxidase demonstrated 10% positivity in untreated tumor (Figure 1c) and 20% positivity in treated tumor (Figure 1f). Consistently, the AMC peak area (arbitrary unit, reflecting capsease-3 activity) in untreated tumor was 460,886 and in treated tumor was 7,234,911 (15.7-fold higher). The AMC peak area in treated tumor decreased to 1,523,682 (79% inhibition) in the presence of the pancaspase inhibitor zVAD, confirming caspases were responsible for the cleavage of Ac-DEVD-AMC (Figure 1 g). The tumor had a cellular respiration rate of 0.17 μM O_2_ min^-1^ mg^-1^ (Figure 1 h). Cytochrome C expression was similar in both treated and untreated tumors, with a positive staining of moderate intensity (2+) in >75% of neoplastic cells (Additional file 1). Annexin A2 expression was 3+ in the untreated tumor and 2+ in the treated tumor (Additional file 2). Thus, this invasive ductal carcinoma demonstrated treatment-associated morphologic and some apoptotic changes (↑caspase-3 activity), Table 1.

**Table 1:** **Clinicopathological features of the studied tumors**

**Tumor**	**Tumor features & outcome**	**Tumor response to the GSK2126458 treatment**				
		**Morphology**	**Caspase-3 activity**	**Immunohistochemistry**		
				**Caspase-3**	**Cytochrome C**	**Annexin A2**
**I**	- Invasive ductal carcinoma, NOS; grade 3; CS IIB; size 4.5 cm	**+**	**+** (↑15.7 fold)	**+** (10% → 20%)	¶ (2+ → 2+)	¶ (3+ → 2+)
	- ER+, PR-, Her2-neu+, Ki-67 = 70%					
	**-** *k*_*c*_ = 0.17 μM O_2_ min^-1^ mg^-1^					
	**-** Age 73 yr; alive at 2 yr with lung and lymph nodes metastases					
**II**	**-** Invasive ductal carcinoma, NOS, NH grade 2; CS IIB; size 4.5 cm	**-**	**+** (↑3.6 fold)	**+** (1% → 3%)	**+** (2+ → 3+)	¶ (2+ → 2+)
	- ER+, PR+, Her2-neu-, Ki-67 = 5%					
	- *k*_*c*_ = 0.15 μM O_2_ min^-1^ mg^-1^					
	- Age 58 yr; free of disease at 18 months					
**III**	**-** Invasive lobular carcinoma, NOS; NH grade 3; and lobular carcinoma in situ (60% of the tumor mass; *the studied component*); CS IIIA; size 6.0 cm	**+**	**-**	**-** (1% → 1%)	¶ (3+ → 3+)	**±** (!) (2+ → 3+)
	**-** ER+, PR+, Her2-neu-, Ki-67 = 30%					
	**-** *k*_*c*_ = 0.22 μM O_2_ min^-1^ mg^-1^					
	- Age 62 yr; free of disease at 18 months					
**IV**	- Invasive ductal carcinoma, NOS; NH grade 2; CS IIB; size 2.3 cm	**+**	**-**	**+** (1% → 2%)	**+** (2+ → 3+)	**+** (2+ → 3+)
	- ER+, PR+, Her2-neu-, Ki-67 = 20%					
	**-** *k*_*c*_ = 0.06 μM O_2_ min^-1^ mg^-1^					
	- Age 41 yr; free of disease at 18 month					
**V**	**-** Ovarian dysgerminoma; CS 1A; size 5.0 cm	**+**	nd	**-**	**ǂ** (3+ → ?)	**ǂ** (2+ → ?)
	**-** *k*_*c*_ = nd					
	**-** Age 22 yr; free of disease at 18 months					
**VI**	- Ovarian serous carcinoma; CS IIIB	**-**	**-**	**-** (1% → 1%)	¶ (3+ → 3+)	¶ (2+ → 2+)
	**-** *k*_*c*_ = nd					
	**-** Age 49 yr; free of disease at 12 months					
**VII**	**-** Moderately differentiated adenocarcinoma, colorectal origin; CS IV	**-**	**-**	**-** (5% → 0%)	**-** (1+ → 1+) (↑Necrosis)	¶ (2+ → 2+)
	**-** *k*_*c*_ = nd					
	**-** Age 60 yr; lost to follow-up					
**VIII**	**-** Poorly differentiated signet ring adenocarcinoma of gastric origin, metastatic to the ovary; CS IV	**-**	**+**	**+** (2% → 4%)	¶ (3+ → 3+)	**+** (2+ → 3+)
	**-** *k*_*c*_ = nd					
	**-** Age 66 yr; advanced disease on palliative care					

*NOS*, Not otherwise specified; *ER*, Estrogen receptor; *PR*, Progesterone receptor; *k*_*c*_, rate of cellular respiration; *CS*, clinical stage; NH grade, Nottingham histological grade; nd, not done due to insufficient tumor sample; ¶, highly positive staining in treated and untreated specimens; !, inconsistent result (Additional file 2); **ǂ**, highly positive staining in untreated specimen (treated specimen was lost). Values in parentheses reflect treatment-associated changes in the expression intensity.

**Figure 2:**
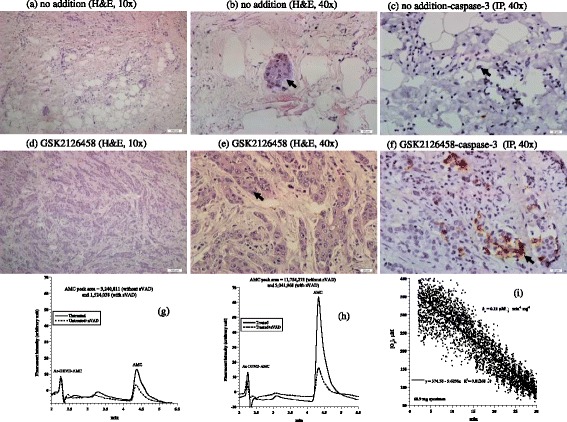
**Breast invasive ductal carcinoma. (a-f)** Histology and expression of caspase-3 by immunoperoxidase. **(a-b)** Untreated tumor demonstrating rare islands of cohesive neoplastic cells (black arrow), H&E at 20× and 40×, respectively. **(d-e)** Treated tumor demonstrating numerous islands and cords of neoplastic cells with a preserved cellular cohesion (black arrow), H&E at 20× and 40×, respectively. **(c)** Untreated tumor demonstrating rare residual neoplastic cells (black arrow) with rare staining for caspase-3 in ~1% of neoplastic cells compared to ~3% in treated tumor **(f)**, immunoperoxidase (IP), 40×. **(g**-**h)** HPLC runs of intracellular caspase-3 activity in treated and untreated tumor. The AMC peak (retention time, ~4.4 min) in treated tumor was blocked by the pancaspase inhibitor zVAD, confirming the cleavage of Ac-DEVD-AMC was mediated by caspases. **(i)** Cellular respiration, measured immediately on tumor arrival to the laboratory to affirm viability.

The second tumor was another invasive ductal carcinoma of the breast (Nottingham histological grade 2). Its hormonal status was ER+, PR+, and Her2-neu-; Ki-67 proliferation index was 5%. Histological features of the treated and untreated specimens were similar (Figure 2 a-b *vs.* d-e). Expression of caspase-3 by immunoperoxidase demonstrated positivity in 1% untreated tumor neoplastic cells (Figure 2c) and 3% positivity in treated tumor (Figure 2f). Intracellular caspase activity was 3.6-fold higher in the treated tumor (Figure 2 g-h). Cytochrome C expression was more prominent in the treated specimen demonstrating an intensity of 3+ in >75% of neoplastic cells compared to the untreated specimen that demonstrated a 2+ intensity of staining in 26-75% of neoplastic cells (Additional file 1). Annexin A2 expression was 2+ in both specimens (Additional file 2). The cellular respiration rate was 0.15 μM O_2_ min^-1^ mg^-1^ (Figure 2i). Thus, only treatment-associated apoptotic changes were evident in this tumor.

**Figure 3:**
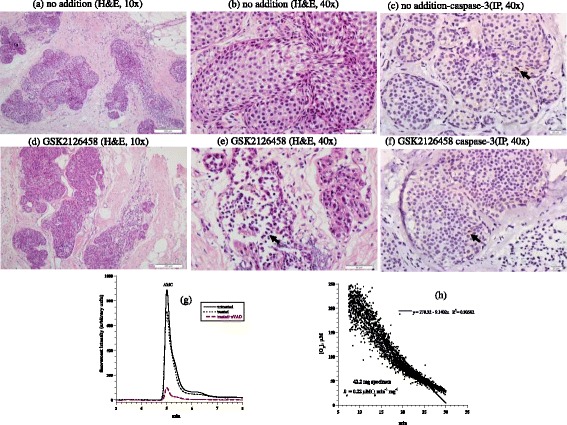
**Breast lobular carcinoma in situ. (a-f)** Histology and expression of caspase-3 by immunoperoxidase. **(a-b)** Untreated tumor demonstrating expanded acini by an in situ proliferation of uniform neoplastic cells, H&E at 10× and 40×, respectively. **(d-e)** Treated in situ carcinoma demonstrating fragmentation and degeneration of neoplastic cells (black arrow), H&E at 10× and 40×, respectively. **(c and f)** Untreated and treated tumor showed rare positivity (1%, black arrow) for caspase-3, immunoperoxidase (IP), 40×. **(g)** HPLC runs of intracellular caspase-3 activity in treated and untreated tumor. The AMC peak (retention time, ~4.8 min) in treated tumor was blocked by zVAD. **(h)** Cellular respiration, measured immediately on tumor arrival to the laboratory to affirm viability.

The third case was an invasive lobular carcinoma of breast (Nottingham histological grade 3). The tumor was ER+, PR+, and Her2-neu-; the Ki-67 proliferation index was 30%. The invasive tumor was associated with an in-situ component that represented about 60% of the tumor. Representative samples of tumor used in this study demonstrated predominantly the in situ carcinoma. Untreated tumor showed cells mostly confined to distended lobular acini by a solid proliferation of relatively uniform poorly cohesive cells. Many of the cells contained small intracytoplasmic vacuoles (Figure 3a-b). Treated tumor demonstrated a decrease in the density of cells with increased cellular dyscohesion and fragmentation of cytoplasm and many degenerative nuclei (Figure 3d-e). Expression of caspase-3 demonstrated 1% positivity in both the treated and untreated tumor (Figure 3c and f). Intracellular caspase activity was also similar in both specimens (Figure 3 g). Cytochrome C (3+ in > 75% of in situ neoplastic cells) was highly expressed in treated and untreated specimens. Annexin A2 was positive in both treated and untreated samples, but showed a higher intensity in treated tumor (2+ in treated tumor compared to 1+ in untreated tumor), Additional files 1 and 2. Cellular respiration rate was 0.22 μM O_2_ min^-1^ mg^-1^ (Figure 3 h). Thus, only treatment-associated morphologic changes were evident in this tumor.

**Figure 4:**
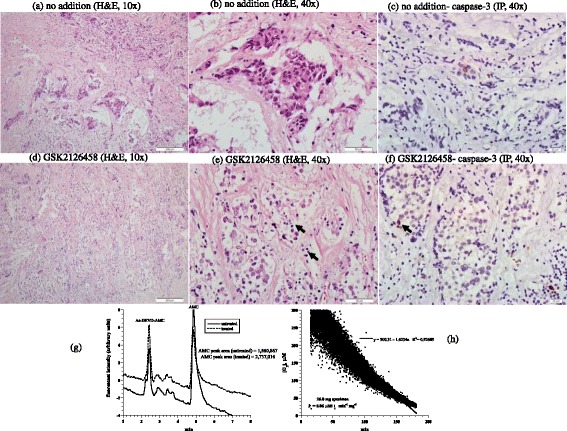
**Invasive ductal carcinoma. (a-f)** Histology and expression of caspase-3 by immunoperoxidase. **(a-b)** Untreated tumor cords and nests of neoplastic cells in a background of desmoplastic reaction, H&E at 10× and 40×, respectively. **(d-e)** Treated tumor demonstrating decreased cellularity, cellular dyscohesion and numerous apoptotic bodies (black arrow), H&E at 10× and 40×, respectively. **(c and f)** demonstrating rare expression by caspase-3 in untreated tumor ( 1%) compared to up to 2% in treated tumor, immunoperoxidase (IP), 40×. **(g)** HPLC runs of intracellular caspase-3 activity in treated and untreated tumor (AMC peak retention time, ~4.8 min). **(h)** Cellular respiration, measured immediately on tumor arrival to the laboratory to affirm viability.

The fourth tumor was invasive ductal carcinoma of the breast (Nottingham histological grade 2). The hormonal status was ER+, PR+, Her2-neu-; the Ki-67 proliferation index was 20%. Histological features of the untreated tumor showed neoplastic cells arranged in cords and nests with moderate nuclear pleomorphism, amphophilic cytoplasm, vesicular nuclei, inconspicuous nucleoli, and a background of desmoplastic reaction (Figure 4a-b). Treated tumor revealed decreased cellular density with dyscohesion and numerous apoptotic bodies, suggesting a morphologic response (Figure 4d-e). Expression of caspase-3 demonstrated 1% positivity in untreated tumor (Figure 4c) and 2% positivity in treated tumor (Figure 4f). Caspase activity was about the same in treated and untreated samples (Figure 4 g). Cytochrome C was positive in both treated and untreated tumor with a moderate intensity; but it was positive in >75% (3+) of cells in treated tumor compared to 26-75% (2+) of cells in the untreated tumor. Annexin A2 expressions was increased in intensity in the treated specimen (3+) compared to the untreated specimen (2+), Additional files 1 and 2. The cellular respiration rate was 0.06 μM O_2_ min^-1^ mg^-1^ (Figure 4 h). Thus, this tumor demonstrated treatment-associated morphologic and apoptotic changes.

**Figure 5:**
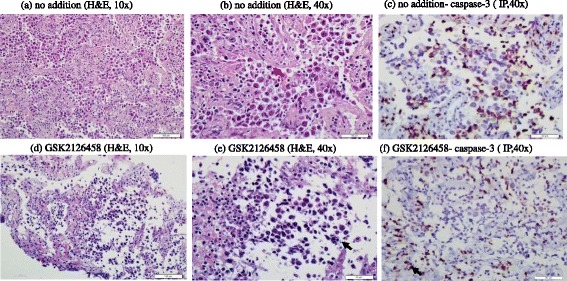
**Ovarian dysgerminoma. (a**-**f)** Histology and expression of caspase-3 by immunoperoxidase in treated and untreated tumor specimens. **(a**-**b)** untreated tumor demonstrated nests of neoplastic cells separated by fibrous bands rich in lympho-plasmacytic infiltrate. Mitotic figures are numerous (red arrow), H&E, 10× and 40×, respectively. **(d**-**e)** Treated tumor revealing decreased cellular density with cytoplasmic disintegration, vacuolar degeneration, nuclear basophilia, pyknotic nuclei, and numerous apoptotic bodies (black arrow), H&E, 10× and 40×, respectively. **(c and f)** Expression of caspase-3 in untreated tumor is seen in lymphocytes (black arrow) and some of neoplastic cells. In the treated tumor, there is positivity in residual lymphocytes and lack of positivity in disintegrating pyknotic neoplastic cells, immunoperoxidase (IP), 40×.

The fifth tumor was ovarian dysgerminoma. Histological features of the untreated tumor revealed nests of neoplastic cells separated by fibrous bands rich in lympho-plasmacytic infiltrate. Neoplastic cells were characterized by uniformly round central nuclei, clear-to-eosinophilic cytoplasm, and prominent single nucleoli. Mitotic figures were abundant (Figure 5a-b). Treated tumor revealed decreased cellular density with cytoplasmic disintegration, vacuolar degeneration, nuclear basophilia, pyknotic nuclei, and numerous apoptotic bodies (Figure 5d-e). Expression of caspase-3 in untreated tumor demonstrated positivity in lymphocytes and some of neoplastic cells (Figure 5c). In treated tumor, there was positivity in residual lymphocytes and lack of positivity in disintegrating pyknotic neoplastic cells (Figure 5f). Assessment of cytochrome C and annexin A2 expression in the untreated tumor was not feasible due to loss of tissue. Thus, this tumor demonstrated only treatment-associated morphologic changes.

**Figure 6:**
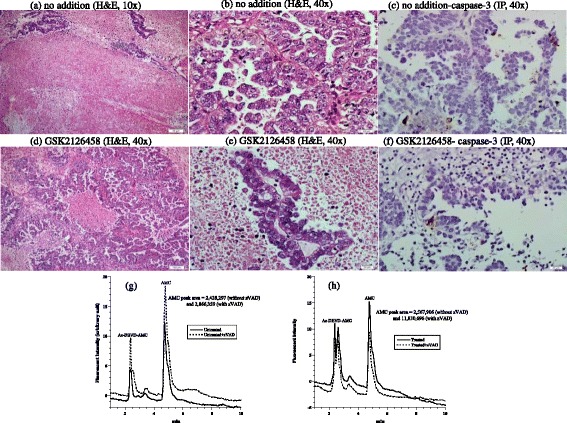
**Ovarian serous carcinoma. (a** **-** **f)** Histology and expression of caspase-3 by immunoperoxidase in treated and untreated tumor specimens. **(a** **-** **b and c** **-** **d)** There is preserved tumor histology in the treated tumor compared to untreated tumor, H&E, 10 and 40×. **(c and f)** stained similar in both tumors with a low level of expression, immunoperoxidase (IP, 40×). **(g** **-** **h)** HPLC runs of intracellular caspase-3 activity in treated and untreated tumor (AMC peak retention time, ~4.8 min).

The sixth tumor was high-grade serous ovarian carcinoma. The untreated tumor consisted of neoplastic cells arranged in papillary folds, slit like fenestrations and complex glands. The cells showed marked nuclear atypia and pleomorphism with frequent mitoses. There was extensive tumor associated necrosis (Figure 6a-b). Treated tumor demonstrated similar histology, but less pronounced foci of necrosis (Figure 6d-e). Expression of caspase-3 by immunoperoxidase demonstrated 1% positivity in both treated and untreated specimens (Figure 6c and f). Cytochrome C expression was highly positive (3+ in >75% of neoplastic cells) in both specimens (Additional file 1). Annexin A2 expression was also the same in both specimens (Additional file 2). Consistently, the AMC peak areas for treated and untreated samples were about the same (Figure 6 g-h). Thus, this serous ovarian carcinoma demonstrated no treatment-associated changes.

**Figure 7:**
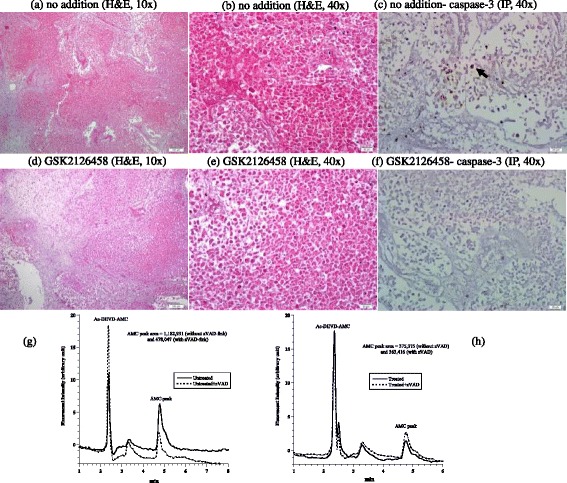
**Metastatic colorectal adenocarcinoma. (a** **-** **f)** Histology and expression of caspase-3 by immunoperoxidase. **(a** **-** **b and c** **-** **d)** Sample consisted of necrotic neoplastic tissue, H&E, 10× and 40×, respectively. **(c and f)** Caspase-3 demonstrated a positive staining (black arrow) in up to 5% of necrotic neoplastic cells in untreated tissue and absence of staining in treated tissue, immunoperoxidase (IP), 40×. **(g** **-** **h)** HPLC runs of intracellular caspase-3 activity in treated and untreated tumor (AMC peak retention time, ~4.8 min).

The seventh tumor was metastatic colorectal carcinoma. Histological examination revealed predominantly necrotic tumor with shadows of neoplastic cells in both treated and untreated samples (Figure 7a-b, d-e). Expression of caspase-3 by immunoperoxidase demonstrated 5% positivity in untreated tumor (Figure 7c) and 0% positivity in treated tumor (Figure 7f). The AMC peak area did not increase in treated tumor (Figure 7 g-h). Cytochrome expression was negative (1+ in <5%) in both specimens with prominent tumor necrosis (Additional file 1). Annexin A2 expression was highly positive (2+) in both treated and untreated specimens (Additional file 2). Thus, assessment of drug effects in this tumor was not feasible as both samples were predominantly necrotic.

**Figure 8:**
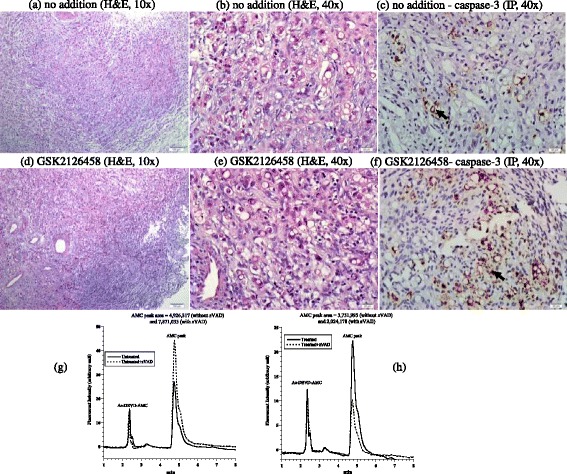
**Metastatic poorly differentiated adenocarcinoma. (a** **-** **f)** Histology and expression of caspase-3 by immunoperoxidase. **(a** **-** **b and c** **-** **d)** Treated and untreated tumors show similar morphological changes, H&E, 10× and 40×, respectively. **(c and f)** Caspase-3 demonstrates a mildly increased staining (black arrow) in treated tumor (4%) compared to (2%) in untreated tumor, immunoperoxidase ( IP), 40×. **(g** **-** **h)** HPLC runs of intracellular caspase-3 activity in treated and untreated tumor (AMC peak retention time, ~4.8 min).

The eighth tumor was a metastatic poorly differentiated adenocarcinoma of gastric origin. Both untreated and treated specimens revealed clusters of neoplastic cells with signet-ring cell morphology. Cytoplasmic and nuclear details were preserved in both samples (Figure 8a-b, d-e). Expression of caspase-3 by immunoperoxidase demonstrated 2% positivity in untreated tumor (Figure 8c) and 4% positivity in treated tumor (Figure 8f). AMC peak area (calculated as area without zVAD minus area with zVAD) in untreated tumor was less than zero and in treated tumor was 1,727,817 (Figure 8 g-h). Cytochrome C was positive in both specimens (>75% of cells, or 3+) (Additional file 1). Annexin A2 expression increased in the treated specimen (Additional file 2). Thus, the GSK2126458 treatment was associated with mild increases in caspase-3 expression and activity.

## Discussion

This study described structural and apoptotic biomarkers associated with exposure of eight relatively viable tumors to the PI3K/mTOR inhibitor GSK2126458. Morphologic changes were observed in four tumors and apoptotic changes in four tumors (Table 1).

Intracellular caspase-3 was assessed by immunoperoxidase staining and by the synthetic cell-permeable caspase-3 substrate Ac-DEVD-AMC. Other utilized apoptotic biomarkers were immunoperoxidase staining for cytochrome C and annexin A2. It is expected that tumor resection will lead to induction of apoptosis with caspase activation. Nevertheless, the GSK2126458 treatment further intensified caspase-3 induction in three tumors (Table 1). This observation is consistent with the recent finding of rapid induction of apoptosis by PI3K inhibitors [18]. It also suggests the suitability of using apoptotic biomarkers to monitor PI3K/mTOR therapeutic activities. It is worth noting, however, that an accurate assessment of the drug effect requires several structural and functional biomarkers (e.g., drug-induced changes in histology, gene expression, caspase induction, and cellular bioenergetics), preferably performed on multiple tumor fragments. It is also imperative to note that *in vitro* observations may not necessarily correlate with patient response to therapy.

Since most tumors are expected to be heterogeneous, the behavior of a fragment may not necessarily represent the entire cell population. This obstacle was somewhat minimized in this study by having the experienced histopathologists sampling multiple areas from each tumor. For example, the majority of Tumor #3 was in-situ carcinoma, with a little invasive lobular carcinoma. This in-situ component probably has less molecular alterations and probably less proliferative potential than the invasive carcinoma. This sampling problem may have reduced caspase activation in the studied tumor component (Figure 3 and Table 1). In tumor # 2, the tissue consisted mostly of fibrous tumor stroma with little neoplastic nests (Figure 2). This artifact hindered adequate assessment of morphologic changes following drug treatment.

The GSK2126458 treatment induced cytotoxicities in the four studied breast tumors (Tumors 1-4). This preliminarily observation supports the role of PI3K in breast carcinogenesis and the potential therapeutic utility of PI3K/mTOR inhibitors in invasive ductal carcinomas [10-13]. The ovarian serous carcinoma (Tumor #6) and adenocarcinomas of colorectal origin (Tumor #7) had high background levels of necrosis and apoptosis; these features hindered an accurate assessment of the treatment-induce changes.

Data on the role of PI3K pathway in ovarian dysgerminoma are limited (Tumor #5). Thus, the observed morphologic changes following GSK2126458 treatment are encouraging and warrant follow-up studies (Figure 5). Unfortunately, we were unable to complete the apoptotic studies in this tumor since the specimen was inadequate (Table 1).

The limitations to this study are recognized. First, the sampled tissue might consist only of necrotic tumor which will affect the study results. This was seen in tumor #7 which consisted predominantly of necrotic tissue. As colorectal carcinoma is known to have large areas of necrotic tissue this explains the presence of necrotic tissue only in the study sample. Necrosis of tumor can be expected if there is a delay in the transportation of tumor after resection from the operating room to the laboratory. Meticulous coordination between the surgeons and pathologists to maintain tumor viability is crucial. Second, the specimens from four tumors were insufficient for performing all planned assessments (Tumors #5-8). Third, there is the issue of tumor heterogeneity, which can affect the results. Experience in the gross identification of different tumor areas in addition to use of multiple samples for each condition facilitates reducing this limitation. Fourth, the assessments were based on single measurements; this inadequacy halted statistical or correlation analyses. Fifth, the study did not include biomarkers for angiogenesis [19] or for blocking PI3K downstream molecules. The described method in current study may not be appropriate for assessing angiogenesis in a given tumor due to the short drug exposure time. Expression of angiogenesis molecules, however, is worth assessing in future studies using this methodology. Sixth, the clinical significance of employed descriptive biomarkers (morphologic and apoptotic changes) remains unclear. These challenges are reasonable goals to overcome in follow-up studies.

## Conclusion

Morphologic derangements and apoptotic intensifications were observed in a few tumors following *in vitro* treatment with GSK2126458 (50 nM for 90 min; e.g., Tumor #1, invasive ductal carcinoma). These preliminarily results confirm that some tumors are highly dependent on the PI3K/mTOR signaling. Further studies are needed to assess the clinical significance of these *in vitro* observations.

## Methods

### Reagents

The PI3K/mTOR inhibitor GSK2126458 (*m.w.* = 505.5, cat. S2658) was purchased from Selleck Chemicals (Houston, TX). The oxygen probe Pd(II) complex of *meso*-tetra-(4-sulfonatophenyl)-tetrabenzoporphyrin **(**Pd phosphor**)** was purchased from Porphyrin Products (Logan, UT). A lyophilized powder of caspase inhibitor I [*N*-benzyloxycarbonyl-val-ala-asp(O-methyl)-fluoromethylketone; zVAD; *m.w* = 467.5; pancaspase inhibitor] was purchased from Calbiochem (La Jolla, CA). The caspase-3 substrate Ac-DEVD-AMC (*N*-acetyl-asp-glu-val-asp-7-amino-4-methylcoumarin; *m.w.* = 675.64; caspase-3 substrate) was purchased from Axxora LLC (San Diego, CA). Rabbit anti-cleaved caspase-3 antibody and rabbit anti-annexin A2 antibody (#D11G2) were purchased from Cell Signaling Technology (Boston, MA, USA). Rabbit anti-cytochrome c antibody [(H-104): sc-7159] was purchased from Santa Cruz Biotechnology, Inc. (Texas, USA). RPMI medium 1640 was purchased from Sigma-Aldrich (St. Louis, MO).

GSK2126458 (9.9 μM), zVAD-fmk (2.14 mM), and Ac-DEVD-AMC (7.4 mM) were reconstituted in dimethyl sulfoxide and stored at -20°C. Pd phosphor (2.5 mg/ml = 2 mM) was reconstituted in dH_2_O and stored in small aliquots at -20°C.

### Tumors

The ethical approval of this study was obtained from Tawam Institutional Review Board and Al Ain Medical District Human Research Ethics Committee (Protocol 11/45). Informed consent was obtained from each patient. Eight tumors were studied (Table 1). The specimens (typically, 0.5×0.5×0.5 cm) were collected immediately following tumor resection, immersed in ice-cold RPMI medium (gassed with 95% O_2_:5% CO_2_), and cut into ~30-mg fragments. Tumor viability was assessed on sample arrival to the laboratory by measuring cellular respiration (cellular mitochondrial O_2_ consumption) as previously described and discussed briefly below [14-17]. Viable tumors were incubated at 37°C in 10 mL RPMI (continuously gassed with 95% O_2_:5% CO_2_) with and without 50 nM GSK2126458 for 90 min (150 min for tumor #5). At the end of the incubation period, the fragments were processed for histology, expression of caspase-3 by immunoperoxidase, and determining intracellular caspase activity using the caspase-3 substrate Ac-DEVD-AMC as previously described and discussed briefly below [14-17].

### Histology

Three micron-thick sections of formalin-fixed, paraffin-embedded tumor tissue were obtained and stained with hematoxylin and eosin (H&E). Positivity for ER, PR, Her2-neu, and Ki-67 was assessed based on clinically utilized guidelines on breast biomarker reporting published by the College of American Pathologist [20].

### Immunohistochemistry

Immunohistochemical detections of caspase-3, cytochrome c, and annexin A2 were performed as previously described [16]. Caspase-3 was expressed as a percentage of tumor cells showing positive nuclear immunostaining. For cytochrome c a dilution of 1:500 was used and for annexin A dilution of 1:200 was used. The immunostaining intensity of cytochrome c was scored using a semi-quantitative manual method. The intensity of staining was scored as: strong (3+), moderate (2+), weak (1+), and negative (0).In addition, the percentage of positive cells was also scored using following: <5% of cells (0), 5–25% (1), 26–75% (2), and >75% (3) of cells. A tumor was regarded as positive if >5% of tumor cells showed immunostaining. A tumor was classified as negative if there was complete absence of immunostaining in tumor cells or if <5% of tumor cells showed positive immunoreactivity [21]. Annexin A2 immunostaining intensity was scored using a semi-quantitative manual method: strong (3+), moderate (2+), weak (1+), and negative (0). The staining pattern was cytoplasmic plus membranous. A positive tumor response to the GSK2126458 treatment was set as at least one scale level increase in the immunostaining intensity [16].

### Caspase activity

Tissue fragments were incubated at 37°C in 1.0 mL RPMI gassed with 95% O_2_:5% CO_2_ with and without 32 μM the pancaspase inhibitor zVAD-fmk for 10 min. The caspase-3 substrate Ac-DEVD-AMC (37 μM) was then added and the incubations continued for 20 min. The fragments were disrupted by vigorous homogenization and 10 passages through a 27-G needle. The supernatants were collected by centrifugation (~16,300 *g* for 90 min) through a Microcentrifuge Filter (nominal *m.w.* limit = 10,000 Dalton, Sigma^©^), separated on HPLC, and analyzed for the free fluorogenic AMC moiety as previously described [14-17]. For tumor #1, the HPLC running solvents were 1:3 CH_3_CN:H_2_O and dH_2_O (1:1 isocratic). For remaining measured tumors, the running solvents were CH_3_OH and dH_2_O (1:1 isocratic).

### Oxygen measurement

Phosphorescence O_2_ analyzer was used to monitor tumor O_2_ consumption at 37°C as previously described [14-17]. Cellular respiration was measured to assess viability of Tumors #1-4 (zero-order kinetics of cellular mitochondrial O_2_ consumption confirms a viable tumor). Briefly, a tumor fragment was placed in 1-mL sealed glass vial and placed in the instrument. O_2_ concentration was monitored as a function of time with the aid of the oxygen probe Pd(II) complex of *meso*-tetra-(4-sulfonatophenyl)-tetrabenzoporphyrin. O_2_ concentration decreased linearly with time, indicating the kinetics of mitochondrial O_2_ consumption was zero-order. The rate of respiration (*k*, μM O_2_ min^-1^) was the negative of the slope d [O_2_]/d*t*. The value of *k* was divided by specimen weight (*k*_*c*_, μM O_2_ min^-1^ mg^-1^).

## Additional files

Additional file 1:
**Immunohistochemical detection of cytochrome C in the studied tumors.** The immunostaining intensity was scored manually: strong (3+), moderate (2+), weak (1+), and negative (0). The following scale was used: <5% of cells (0), 5–25% (1), 26–75% (2), and >75% (3) of cells. A tumor was regarded as positive if >5% of tumor cells showed immunostaining. A tumor was classified as negative if there was complete absence of immunostaining in tumor cells or if <5% of tumor cells showed positive immunoreactivity as previously described [22]. Additional file 2:
**Immunohistochemical detection of annexin A2 in the studied tumors.** The staining pattern is cytoplasmic + membranous. Two antibody dilutions were used (1:200 and 1:400). The immunostaining intensity was scored using a semi-quantitative manual method: strong (3+), moderate (2+), weak (1+), and negative (0).

## Abbreviations

Ac-DEVD-AMC: *N*-acetyl-asp-glu-val-asp-7-amino-4-methylcoumarin
AMC: 7-Amino-4-methylcoumarin
ER: Estrogen receptor
H&E: Hematoxylin and eosin
Her2-neu: Human epidermal growth factor receptor 2-neu
mTOR: Mammalian target of rapamycin 1/2 complexes
Pd: Palladium
PI3K: Phosphatidylinositol-3′ kinase
PR: Progesterone receptor
