# Supplementary material for: In vitro assessment of antitumor activities of the PI3K/mTOR inhibitor GSK2126458
Source: Cancer Cell Int. 2014 Sep 24;14:90. doi: 10.1186/s12935-014-0090-z (PMC4189195; doi:10.1186/s12935-014-0090-z)
Supplement: Additional file 1: — Immunohistochemical detection of cytochrome C in the studied tumors. The immunostaining intensity was scored manually: strong (3+), moderate (2+), weak (1+), and negative (0). The following scale was used: <5% of cells (0), 5–25% (1), 26–75% (2), and >75% (3) of cells. A tumor was regarded as positive if >5% of tumor cells showed immunostaining. A tumor was classified as negative if there was complete absence of immunostaining in tumor cells or if <5% of tumor cells showed positive immunoreactivity as previously described [22]. [file 12935_2014_90_MOESM1_ESM.doc]

| **Additional file 1 Immunohistochemical detection of cytochrome C in the studied tumors** | | |
| --- | --- | --- |
|  | Untreated | Treated |
| **Tumor I**  **Invasive ductal carcinoma** | 2+ in > 75% of neoplastic cells  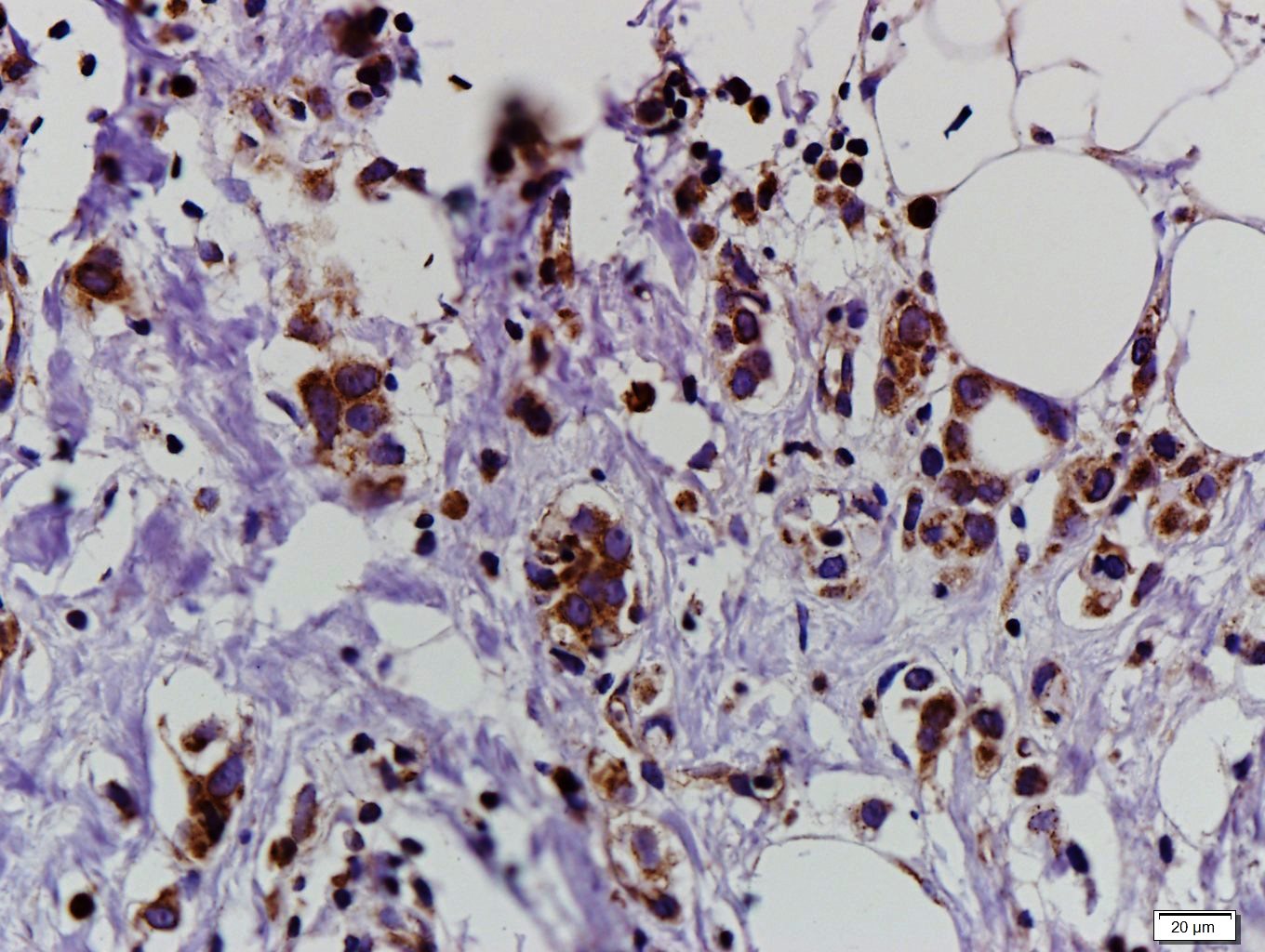 | 2+ in > 75% of neoplastic cells  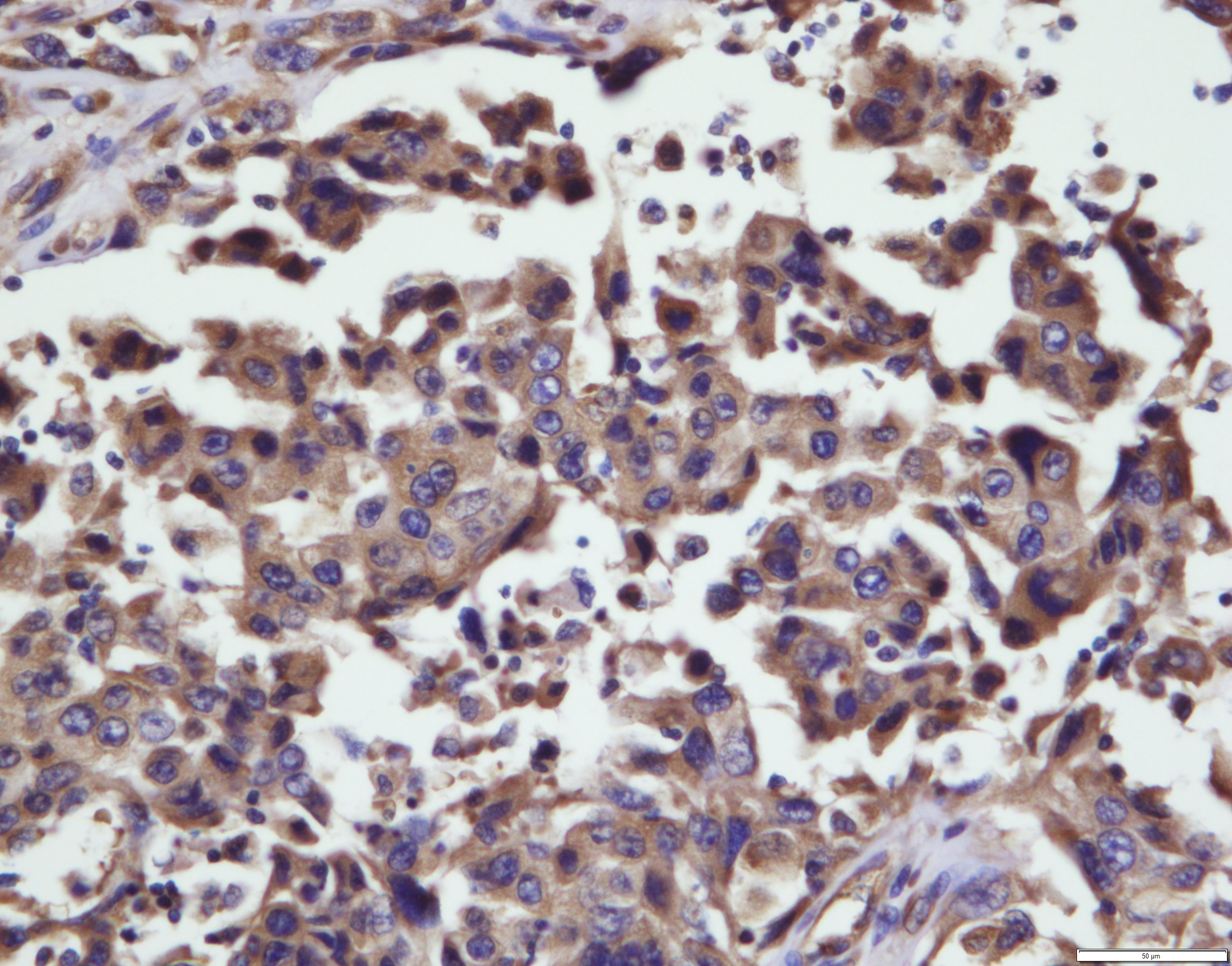 |
| **Tumor II**  **Invasive ductal carcinoma** | 2+ in 26-75% of neoplastic cells  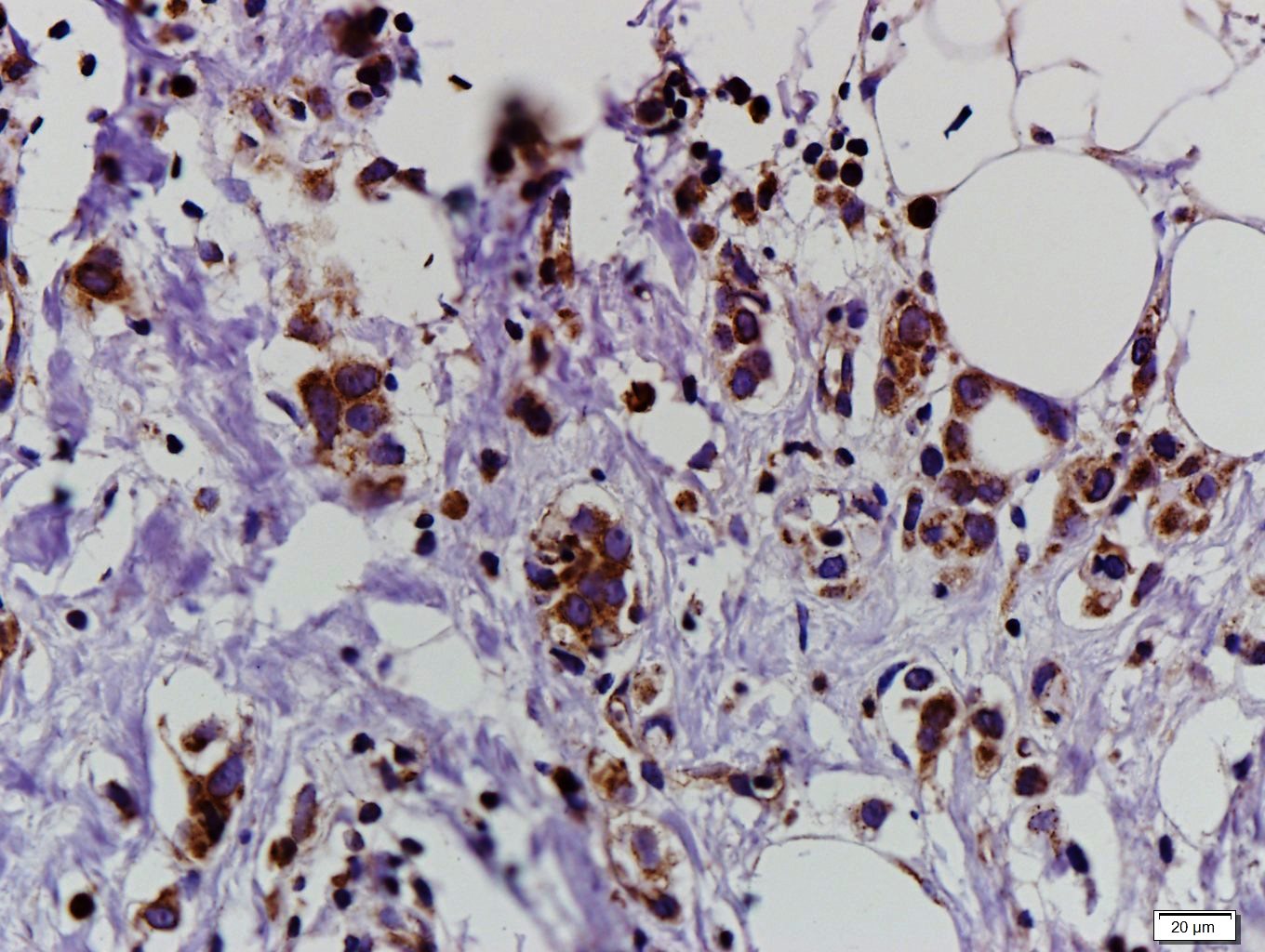 | 3+ in >75% of neoplastic cells  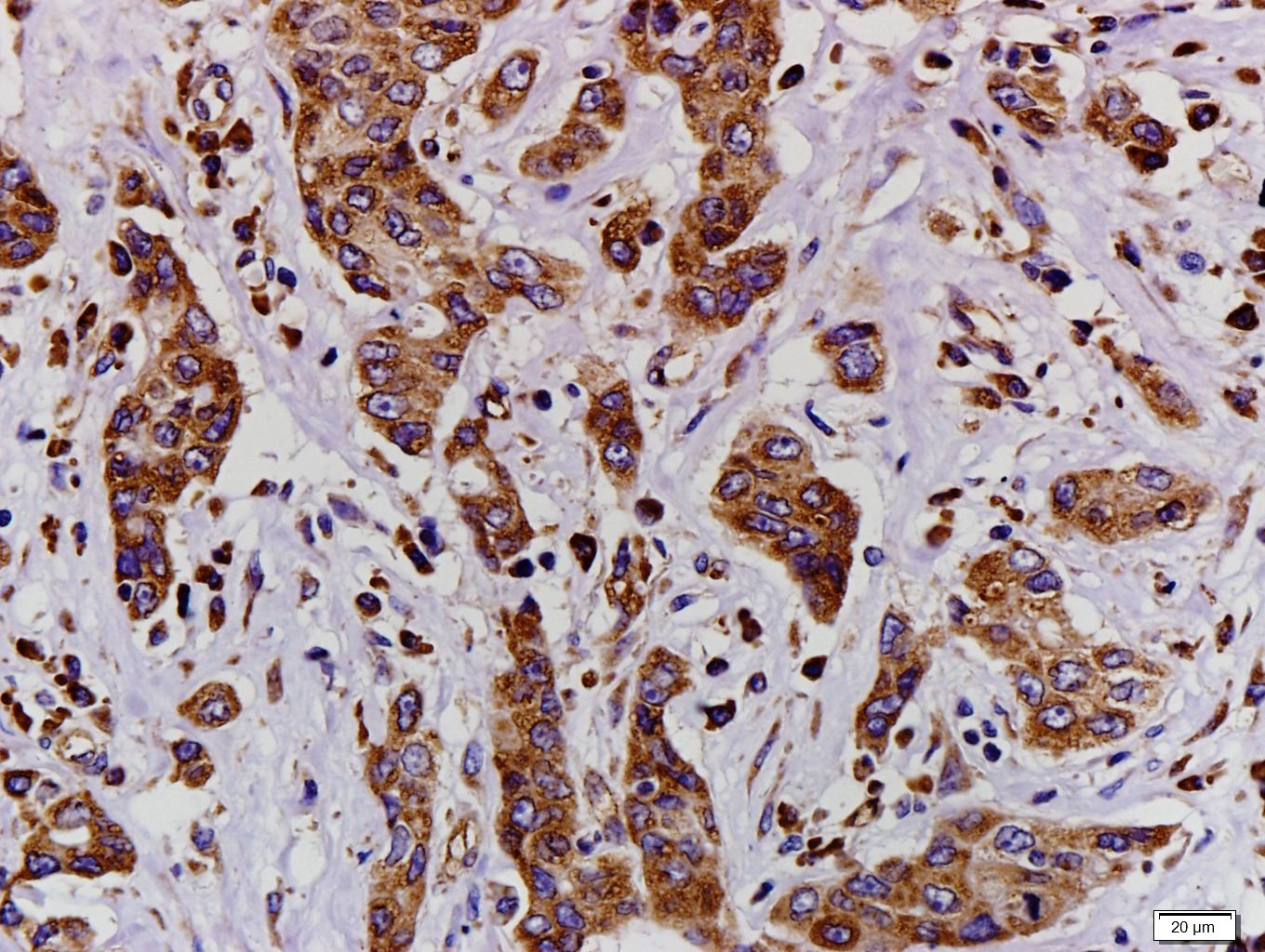 |
| **Tumor III**  **Invasive lobular carcinoma** | 3+ in >75% of neoplastic cells  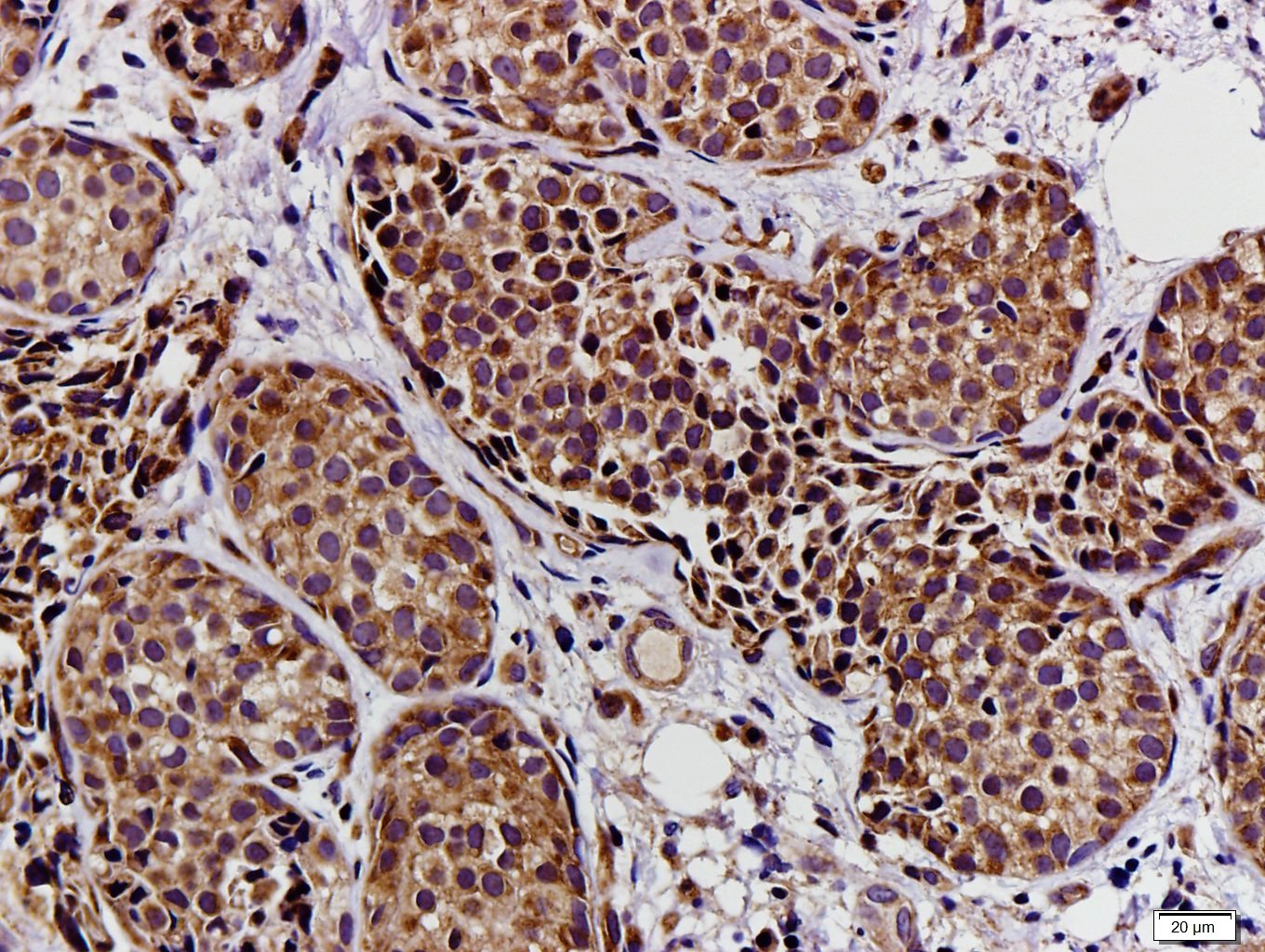 | 3+ in >75% of neoplastic cells  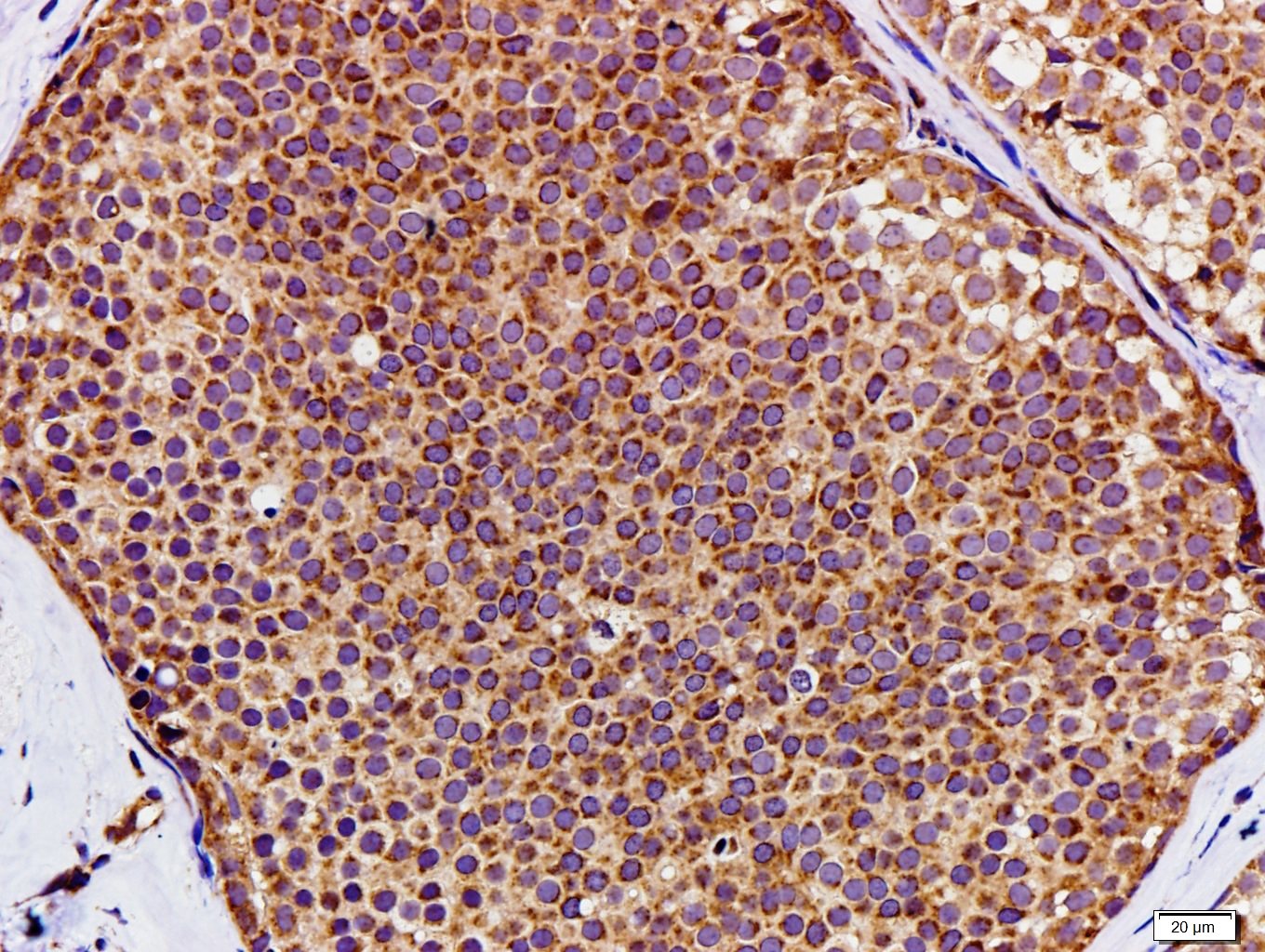 |
| **Tumor IV**  **Invasive ductal carcinoma** | 2+ positivity in 26-75%  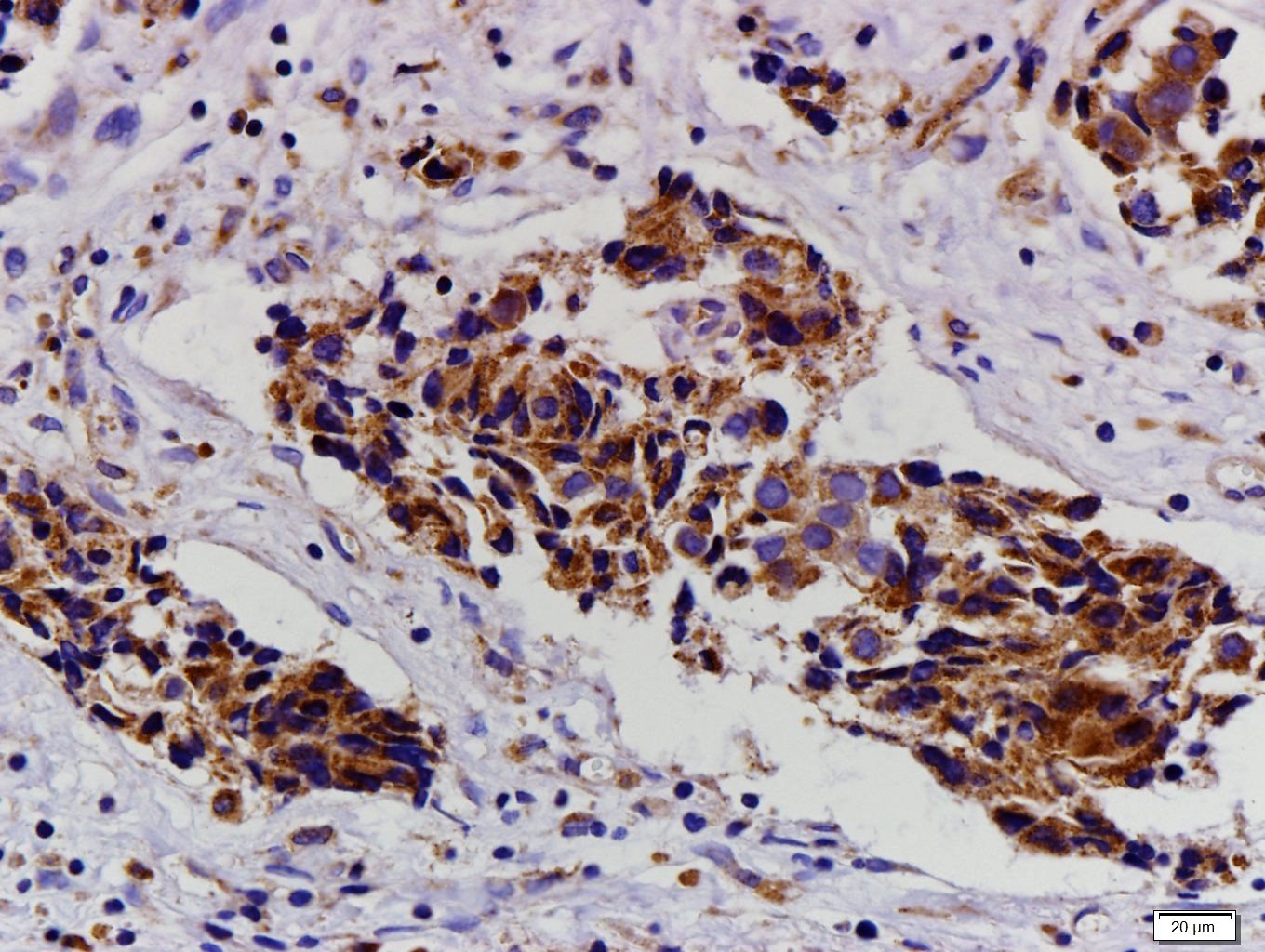 | 2+ positivity in >75%  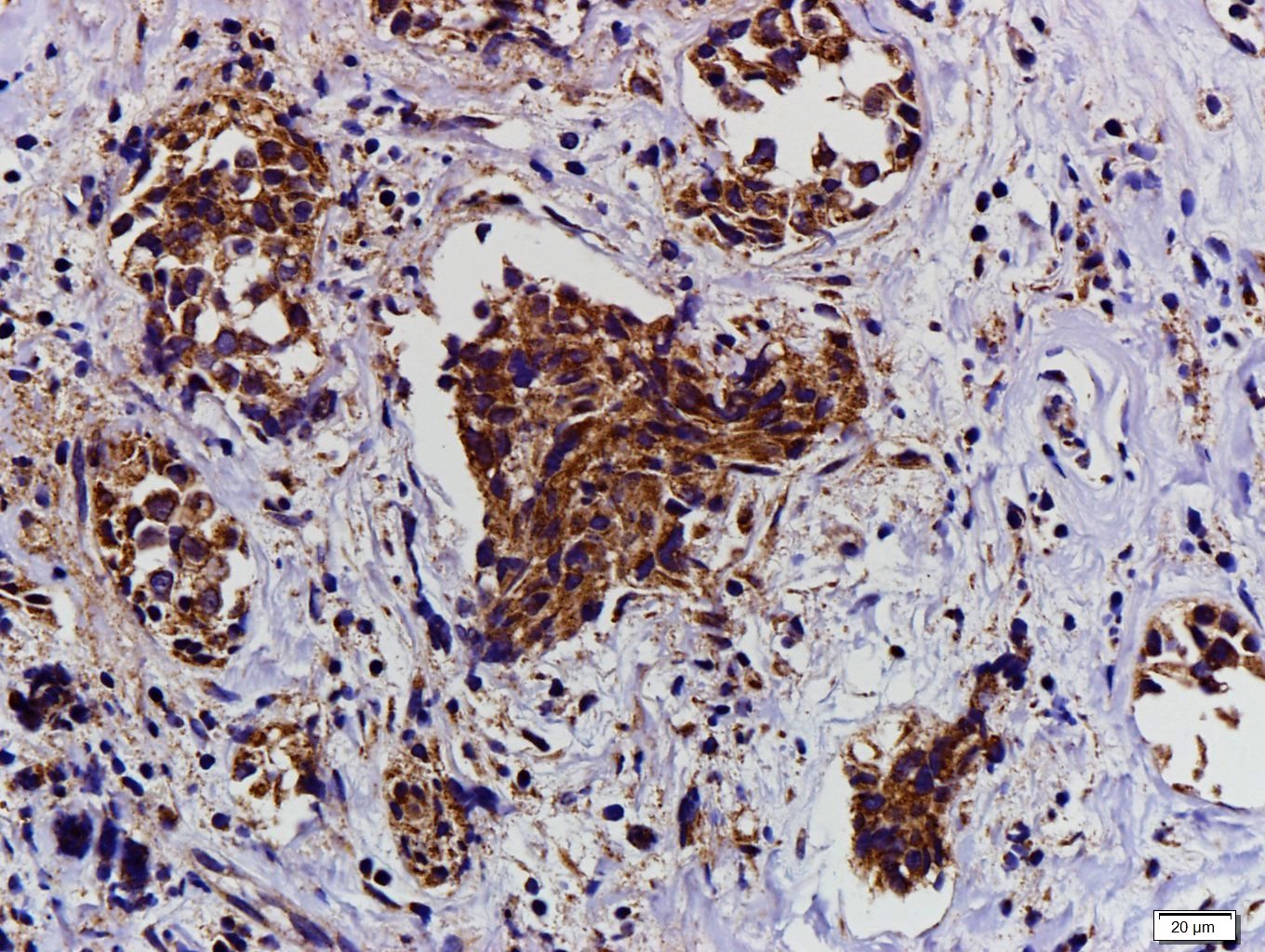 |
| **Tumor V**  **Ovarian dysgerminoma** | 3+ in 26-75% of neoplastic cells  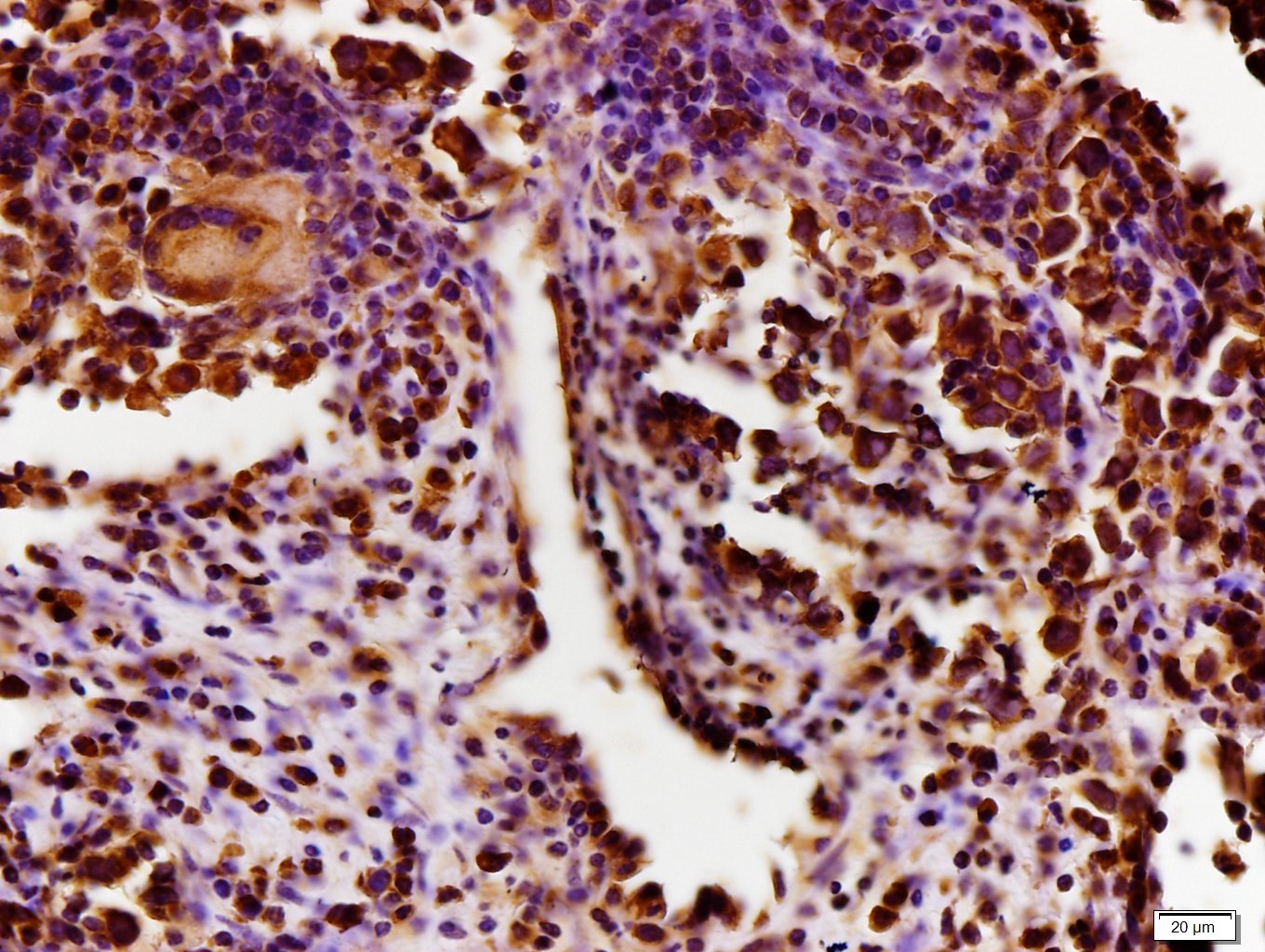 | Tissue lost in the treated condition |
| **Tumor VI**  **Ovarian serous carcinoma** | 3+ in >75% of neoplastic cells  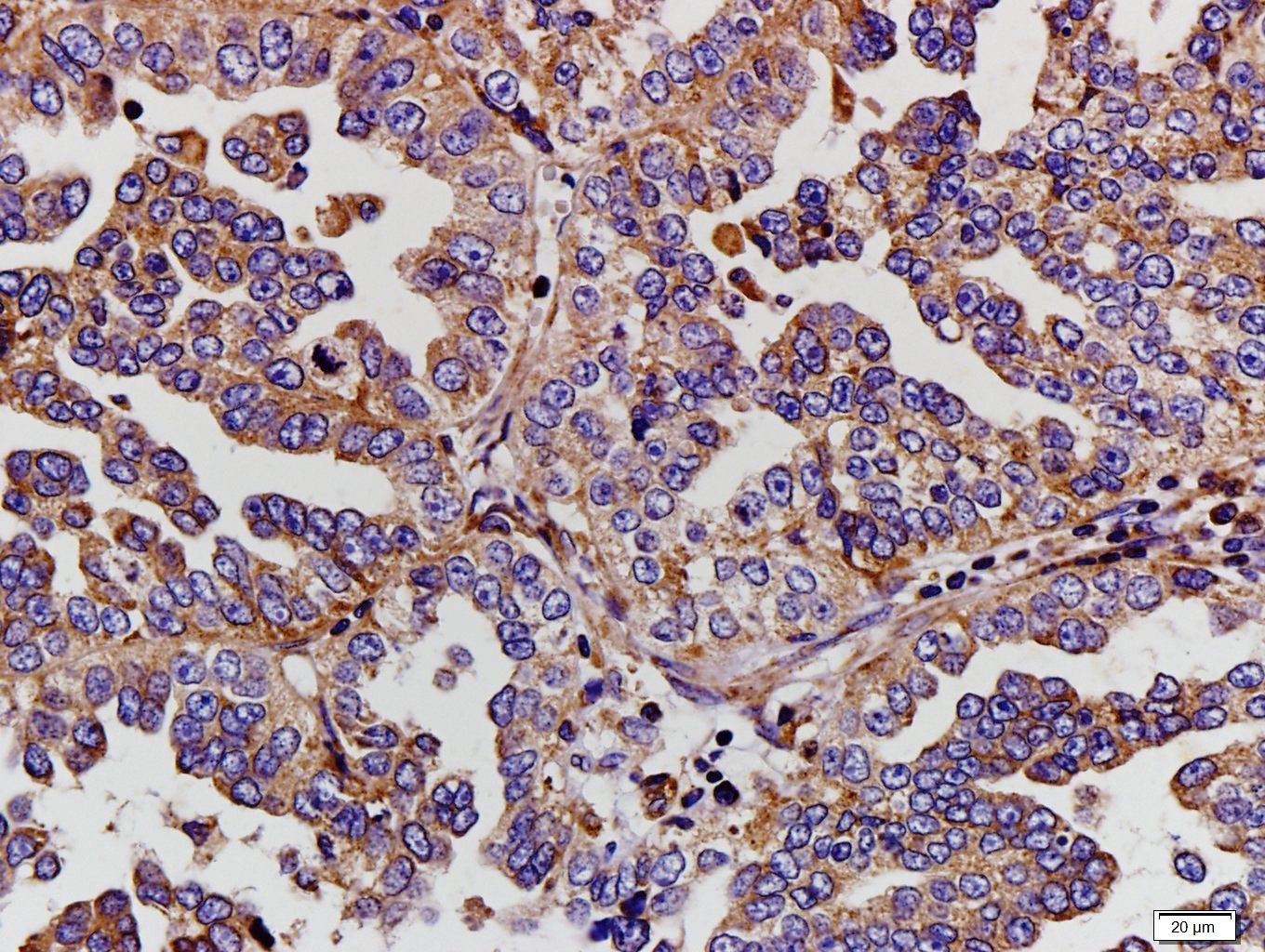 | 3+ in >75% of neoplastic cells  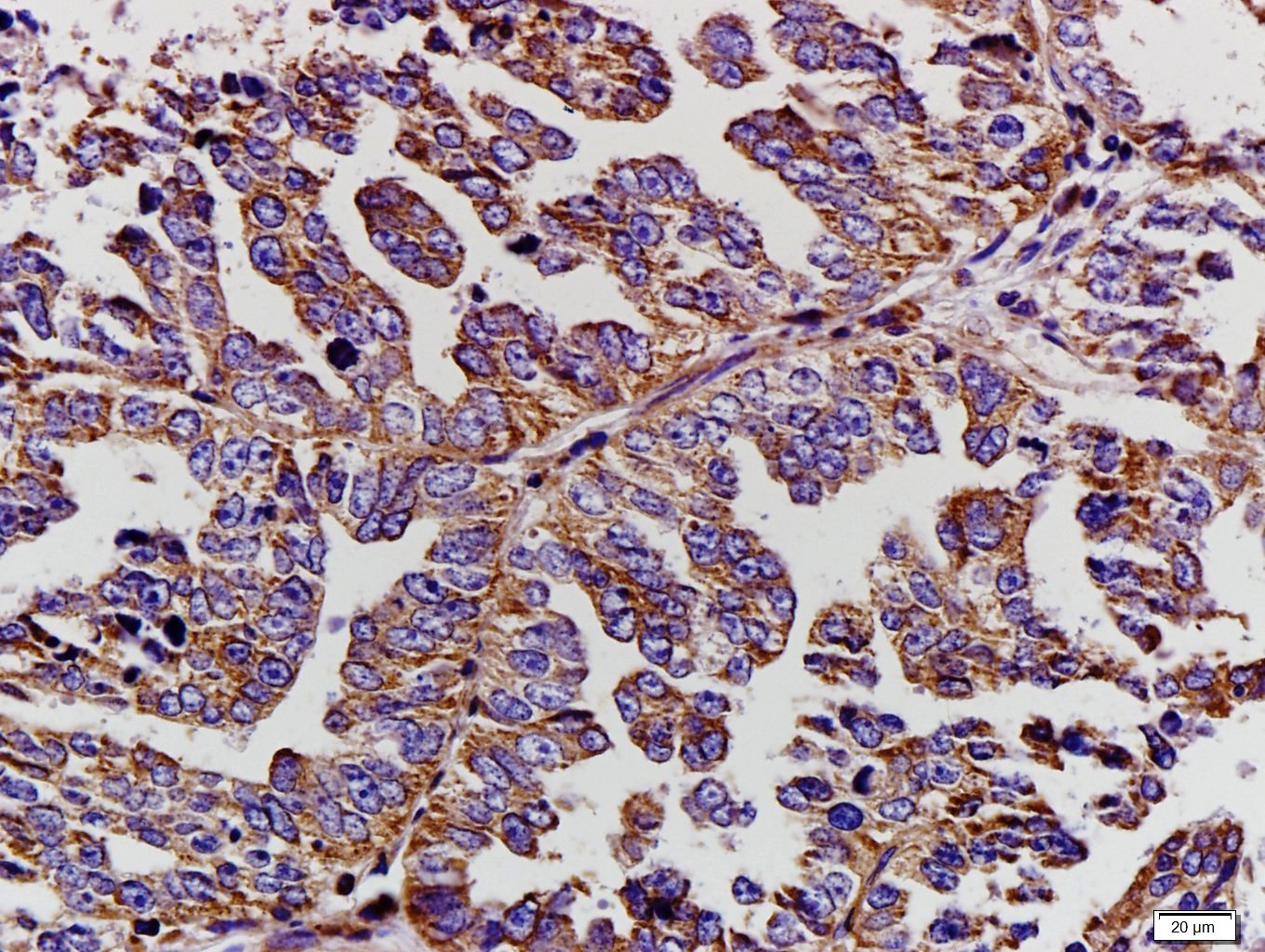 |
| **Tumor VII**  **(necrotic tumor)**  **Moderately differentiated adenocarcinoma of colorectal origin** | Negative: 1+ in <5%  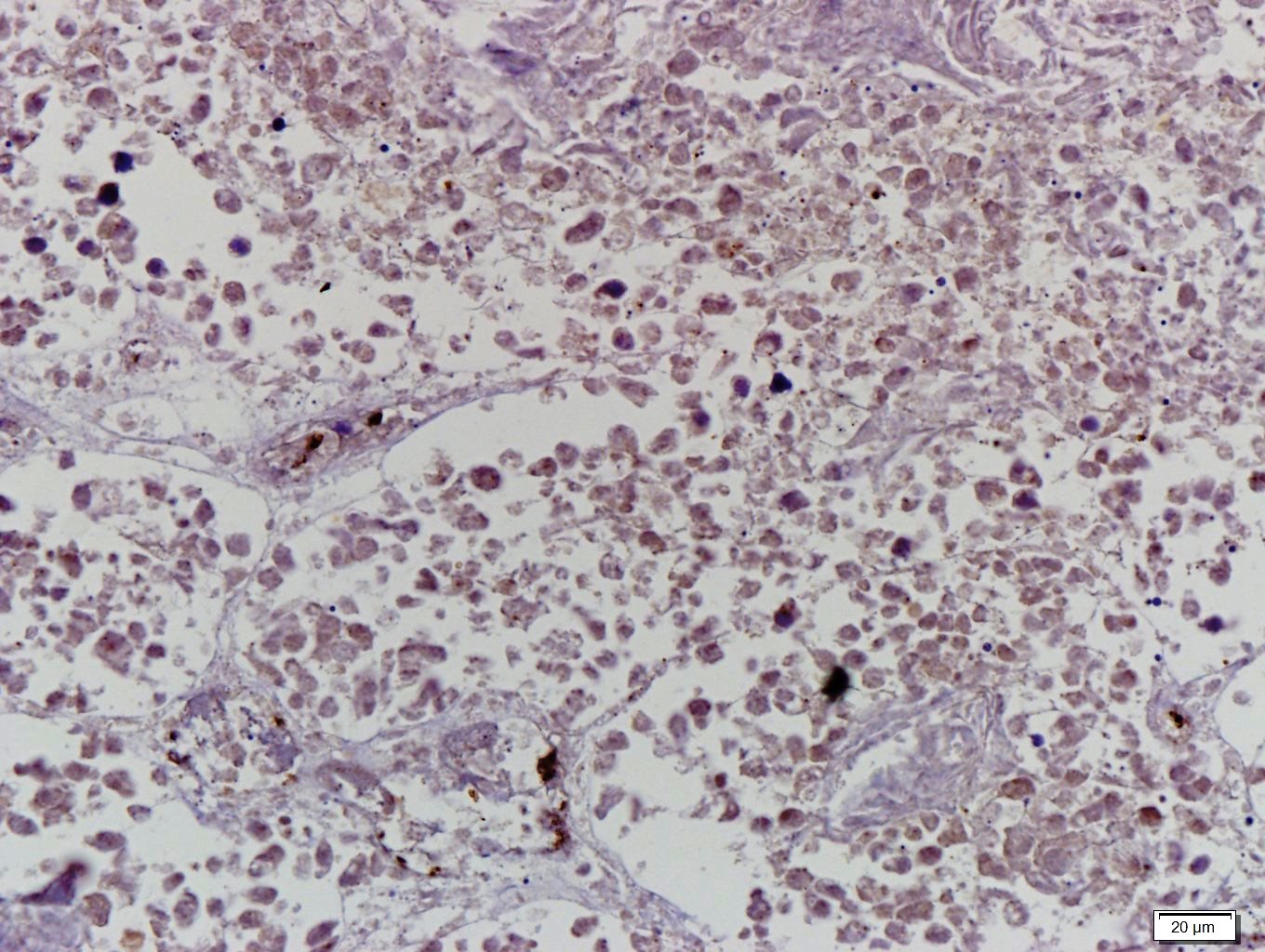 | Negative: 1+ in <5%  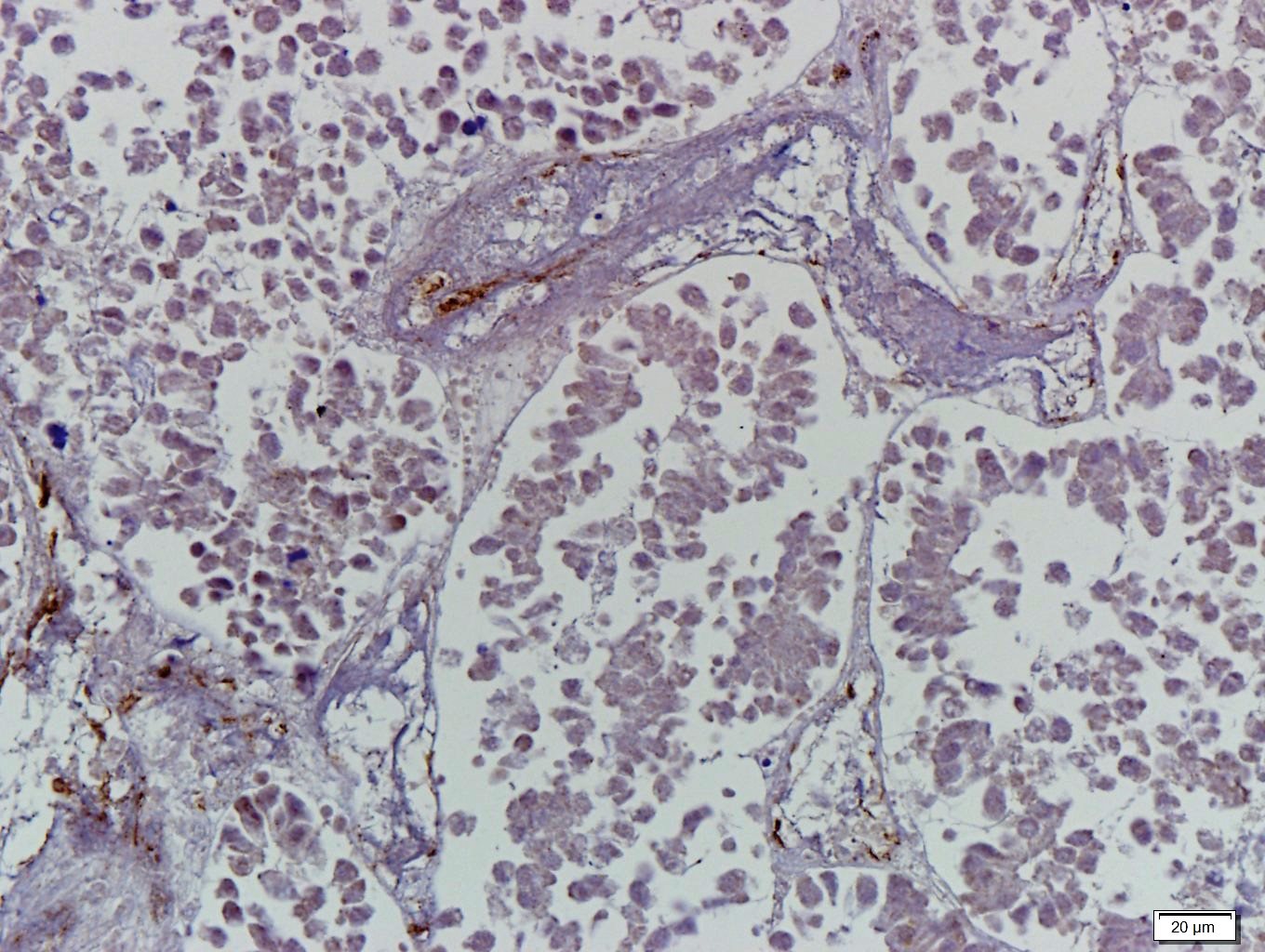 |
| **Tumor VIII**  **Poorly differentiated signet ring adenocarcinoma of gastric origin** | 2+ in >75% of neoplastic cells  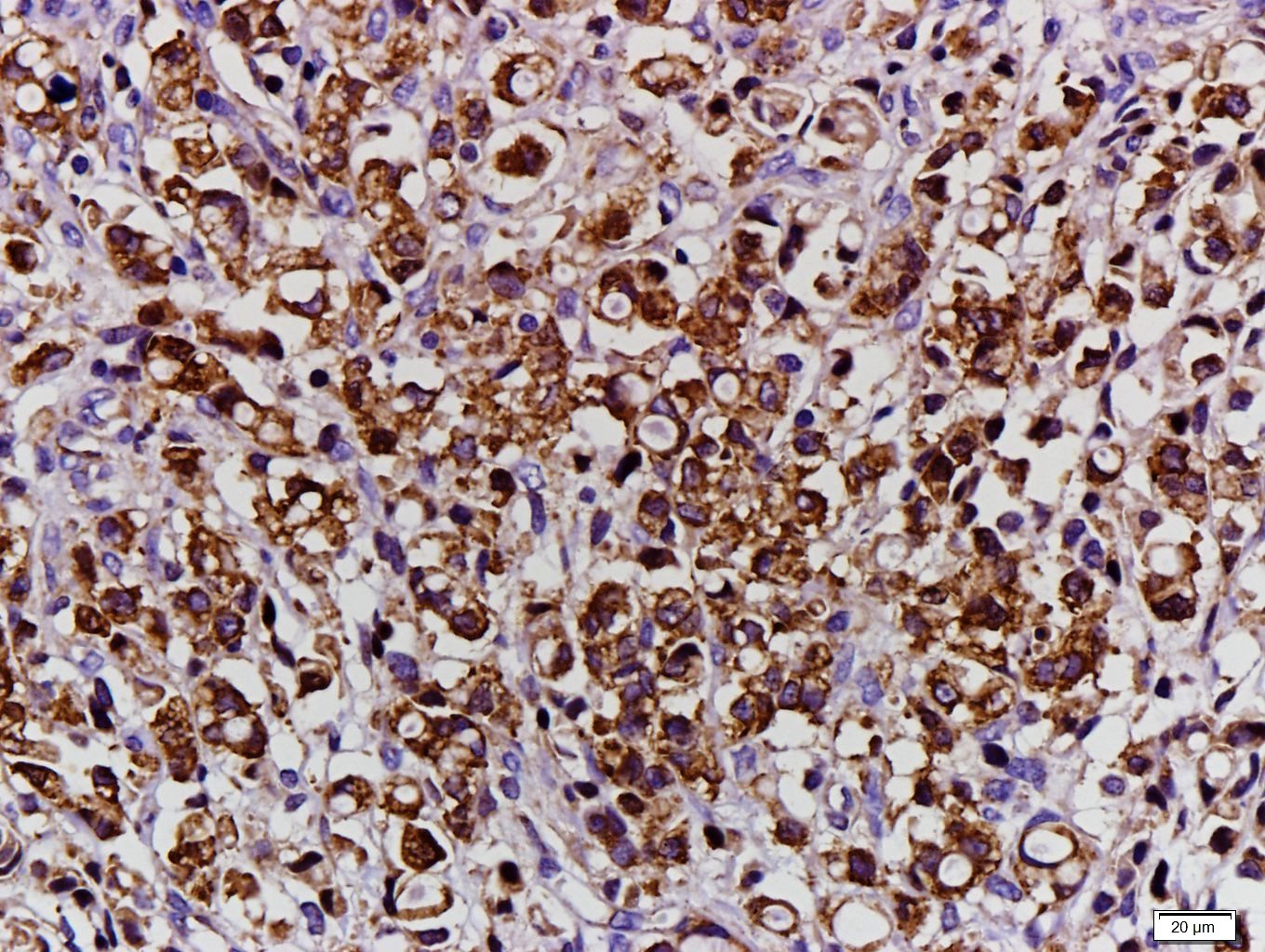 | 3+ in >75% of neoplastic cells  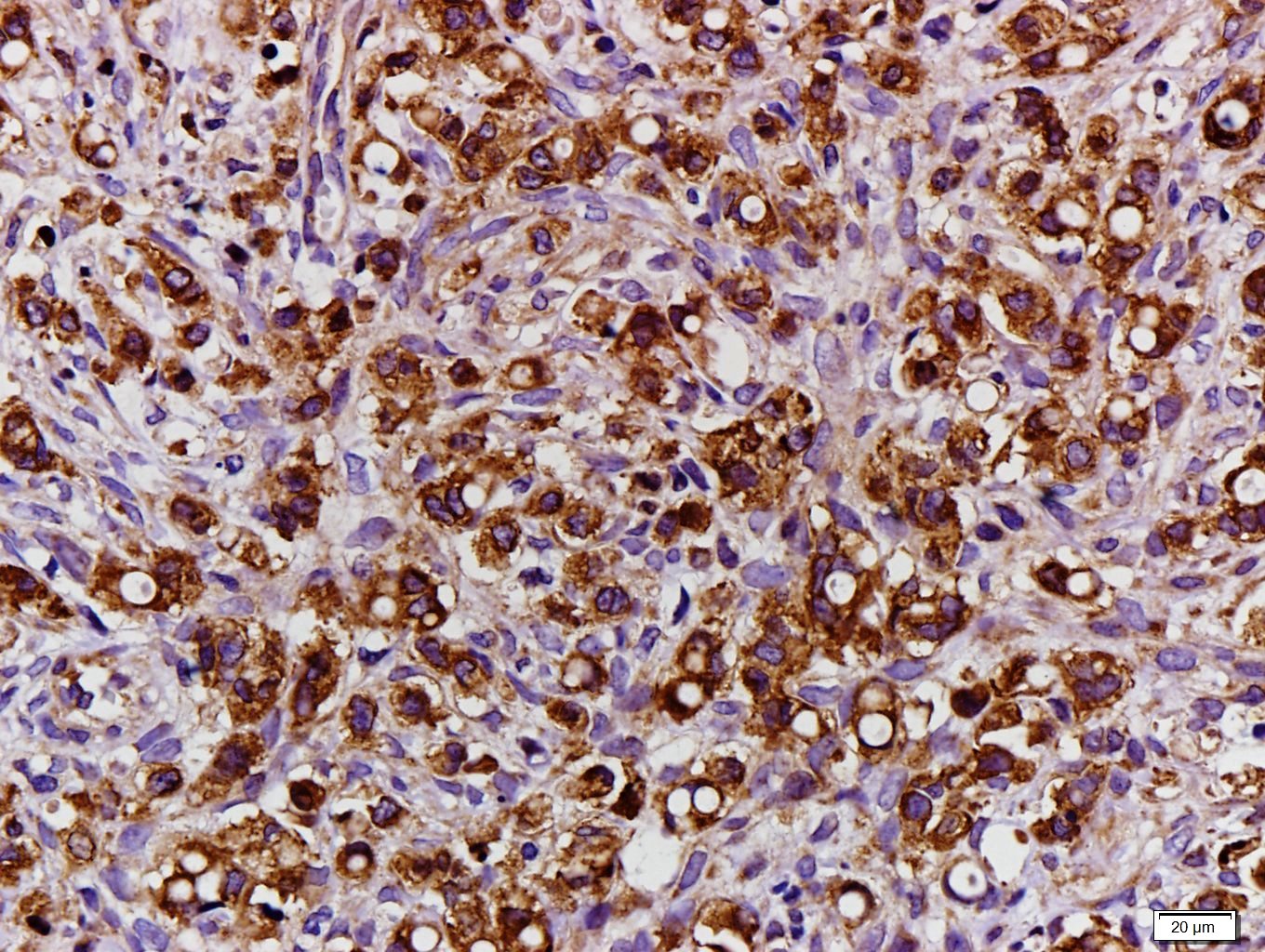 |

Immunostaining intensity was scored using a semi-quantitative manual method: strong (3+), moderate (2+), weak (1+), and negative (0).

The following scale was used: <5% of cells (0), 5–25% (1), 26–75% (2), and >75% (3) of cells. A tumor was regarded as positive if > 5% of tumor cells showed immunostaining. A tumor was classified as negative if there was complete absence of immunostaining in tumor cells or if <5% of tumor cells showed positive immunoreactivity [22].
