# Supplementary material for: In vitro assessment of antitumor activities of the PI3K/mTOR inhibitor GSK2126458
Source: Cancer Cell Int. 2014 Sep 24;14:90. doi: 10.1186/s12935-014-0090-z (PMC4189195; doi:10.1186/s12935-014-0090-z)
Supplement: Additional file 2: — Immunohistochemical detection of annexin A2 in the studied tumors. The staining pattern is cytoplasmic + membranous. Two antibody dilutions were used (1:200 and 1:400). The immunostaining intensity was scored using a semi-quantitative manual method: strong (3+), moderate (2+), weak (1+), and negative (0). [file 12935_2014_90_MOESM2_ESM.docx]

| Additional file 2 **Immunohistochemical detection of annexin A2 in the studied tumors** | | |
| --- | --- | --- |
|  | Untreated ( 1:200 dilution) | Treated ( 1:200 dilution) |
| **Tumor I**  **Invasive ductal carcinoma** | 3+  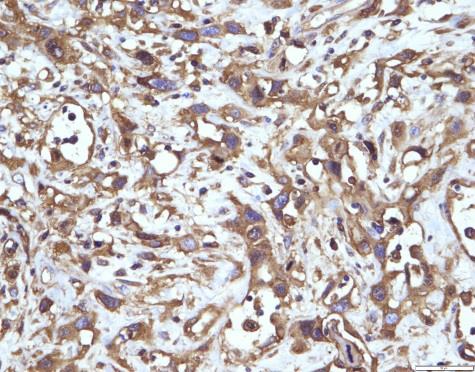 | 2+  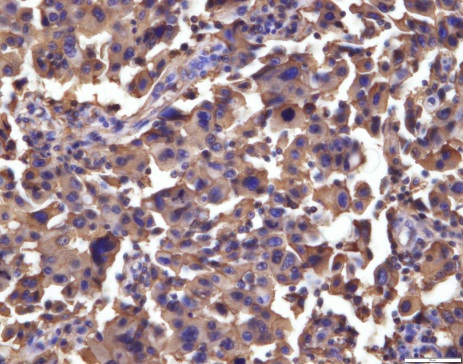 |
| **Tumor II**  **Invasive ductal carcinoma** | 2+  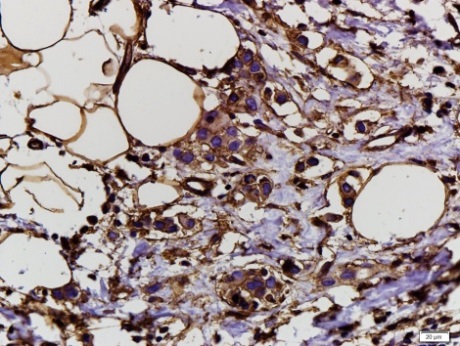 | 2+  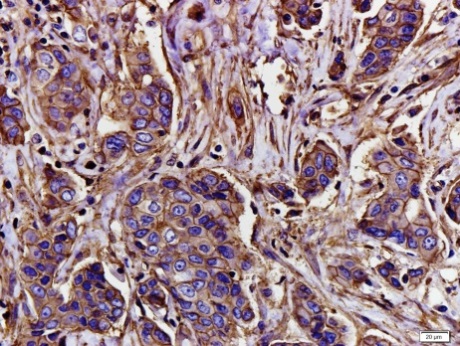 |
| **Tumor III**  **Lobular carcinoma in situ** | 1+  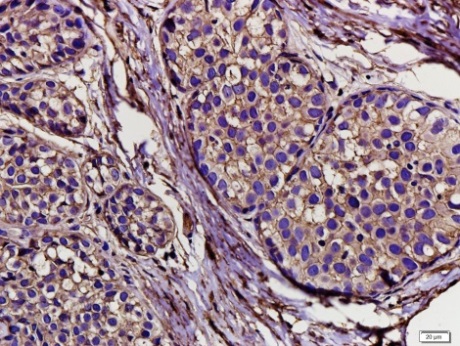 | 2+  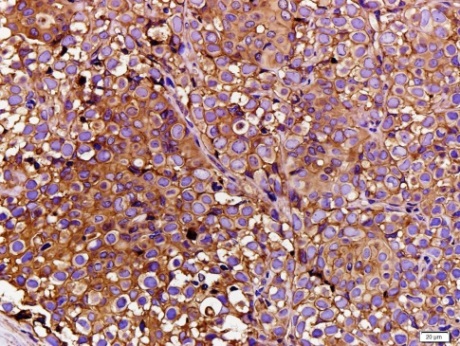 |
| **Tumor IV**  **Invasive ductal carcinoma** | **2+**  **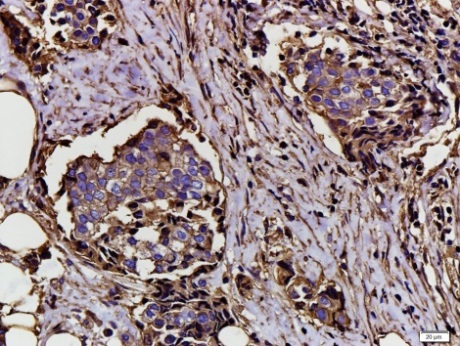** | **3+**  **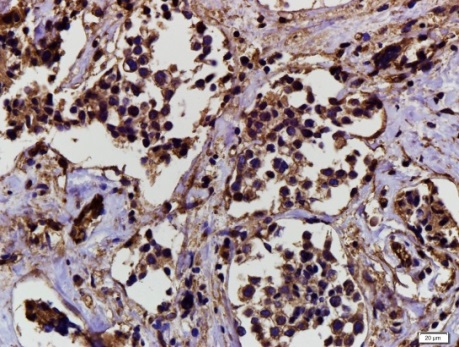** |
| **Tumor V**  **Ovarian dysgerminoma** | 2+ 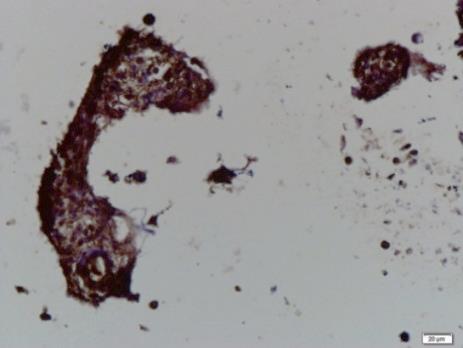 | Tissue lost in the treated conditions |
| **Tumor VI**  **Ovarian serous carcinoma** | 2+  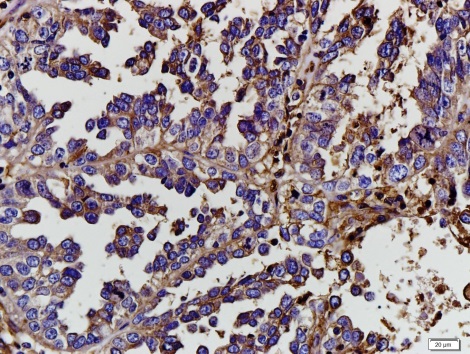 | 2+ 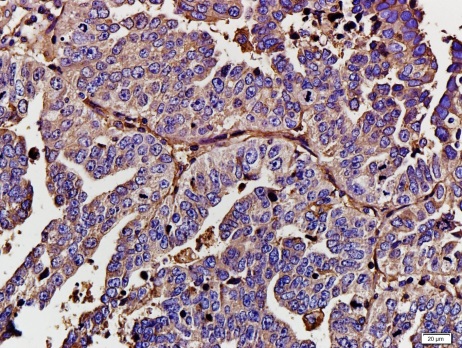 |
| **Tumor VII**  **Moderately differentiated adenocarcinoma of colorectal origin** | 2+  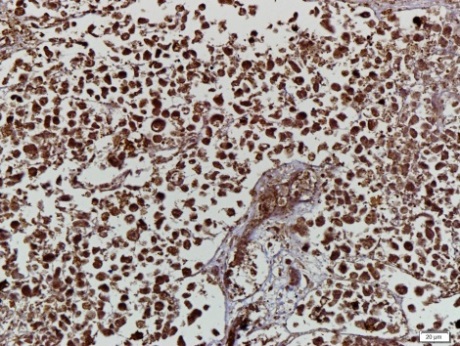 | 2+ 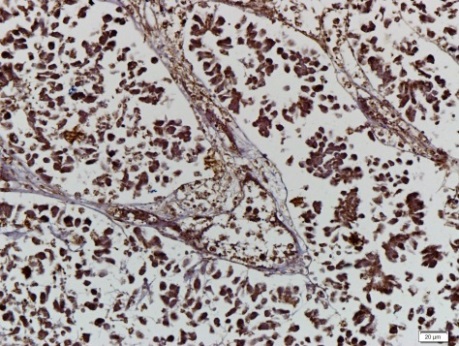 |
| **Tumor VIII**  **Poorly differentiated signet ring adenocarcinoma of gastric origin** | 2+  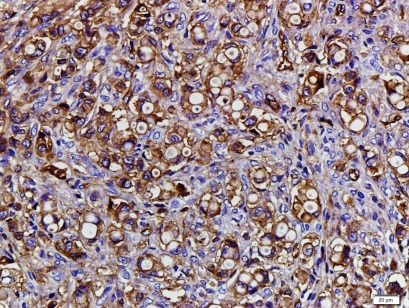 | 3+ 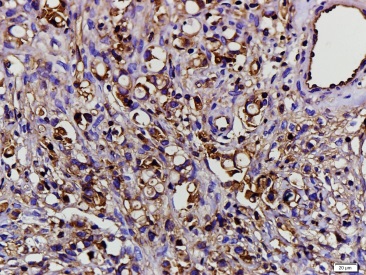 |

The staining pattern is cytoplasmic + membranous. A 1:200 antibody dilution was used. The immunostaining intensity was scored using a semi-quantitative manual method: strong (3+), moderate (2+), weak (1+), and negative (0).
